# Enhancing the thermotolerance and erythritol production of *Yarrowia lipolytica* by introducing heat-resistant devices

**DOI:** 10.3389/fbioe.2023.1108653

**Published:** 2023-02-09

**Authors:** Peixin Liang, Jing Li, Qinhong Wang, Zongjie Dai

**Affiliations:** ^1^ Tianjin Institute of Industrial Biotechnology, Chinese Academy of Sciences, Tianjin, China; ^2^ National Center of Technology Innovation for Synthetic Biology, Tianjin, China; ^3^ College of Biotechnology, Tianjin University of Science and Technology, Tianjin, China

**Keywords:** thermotolerance, yarrowia lipolytica, erythritol, heat-resistant devices, ROS, antioxidant properties

## Abstract

*Yarrowia lipolytica* has been widely used in the food biotech-related industry, where it plays the host’s role in producing erythritol. Nevertheless, a temperature of about 28°C–30°C has been estimated as the yeast’s optimal growth temperature, leading to the consumption of a considerable quantity of cooling water, especially in summer, which is obligatory for fermentation. Herein is described a method for improving the thermotolerance and erythritol production efficiency at high temperatures of *Y. lipolytica*. Through screening and testing different heat resistant devices, eight refactored engineered strains showed better growth at higher temperature and the antioxidant properties of the eight engineered strains were also improved. In addition, the erythritol titer, yield and productivity of the strain FOS11-Ctt1 represented the best among the eight strains, reaching at 39.25 g/L, 0.348 g/g glucose, and 0.55 g/L/h respectively, which were increased by 156%, 86% and 161% compared with the control strain, respectively. This study provides insight into an effective heat-resistant device that could enhance the thermotolerance and erythritol production of *Y. lipolytica*, which might be considered a valued scientific reference for other resistant strains’ construction.

## 1 Introduction

*Yarrowia lipolytica* has been broadly employed in the food-related industry due to its traits of the utilization of inexpensive substrates, high cell density fermentation, and biosafety, especially in the industrial production of the sugar alcohol named erythritol (Fickers et al., 2020; Abbasi et al., 2021). Sources claim that the total erythritol production value is estimated to exceed 150 million USD in 2023. This number corresponds to an increase of approximately 81 million USD compared to the recorded numbers for the year 2017 (Ross, 2018). On the other hand, a temperature of 28°C–30°C has been suggested to be the optimal temperature interval for yeast growth. Additionally, aerobic fermentation requires a large amount of oxygen, which then generates a large amount of biothermal and mechanical heat during the industrial production process (Schenk et al., 2017). Therefore, to avoid the metabolic decline and the risk of bacterial infection caused by excessive temperature (>32°C) (Costa et al., 2014), it is of extreme importance to consume large quantities of cooling water. However, the latter substantially increases the cost of erythritol’s large-scale industrial production.

Recently, adaptive evolution and random mutagenesis found a broad application in tolerance engineering with the ultimate goal of creating industrial strains with superior performance. For example, Qiu et al. identified four genes of the thiamine metabolic pathway that seem to be responsible for the thermostable phenotype of an evolutionarily adaptive mutation in the strain of *Y. lipolytica* employing transcriptomic analysis. They reported a rise in the fermentation temperature of about 3°C, reaching 33°C after overexpression of these genes in original strain (Qiu et al., 2021). Previous work successfully screened out an erythritol hyper producer in the benchtop fermenter at 35°C by combining the ARTP mutagenesis and synthetic microbial biosensor (SMB) system (Qiu et al., 2020). These semi-rational design strategies can produce strains with improved fermentation performance, but the resulting phenotypes are difficult to be transferred to new strains.

Elucidation of the thermal response and defence mechanism of the model yeast *Saccharomyces cerevisiae* has provided valuable targets for the rational breeding of thermotolerant strains. Reports suggest several target molecules have been involved in thermotolerance, including heat shock protein (Hsp), ATPase, trehalose, ubiquitin and antioxidant enzymes, whose role in heat-resistant processes for yeast species has been regarded as vital. Concerning Hsp104, it has proven essential for thermotolerance in *S. cerevisiae*. Sanchez et al. discovered an interval of 100- to 1000-fold difference, accompanied by a temperature increase of 44°C in the *Hsp104Δ* and wild-type cells’ viability (Sanchez et al., 1992). Pma1 in yeast species is one of the most well-known ATPases. Authors have confirmed its pivotal impact on maintaining cells’ structural integrity (Mason et al., 2014) and thermotolerance. The last discovery was based on the significant decrease in the resistance of mutant yeast in which a reduction in PMA1 expression was observed at temperatures like 50°C. The Hul5 ubiquitin ligase was also reported to account for maintaining cell fitness *via* the degradation of misfolded proteins with a short lifetime (Fang et al., 2011). Furthermore, Shahsavarani and colleagues confirmed a positive correlation between overexpression of the *S. cerevisiae* ubiquitin ligase Rsp5 and the increase of the yeast’s upper limit of thermotolerance (Shahsavarani et al., 2012). When ubiquitin ligase Rsp5 was expressed in *Y. lipolytica*, the findings demonstrated a healthy growth of the engineered strain at 34°C and an efficient production of erythritol at 33°C (Wang et al., 2020).

In addition, heat-inducible genes and thermozymes are characteristic of thermophiles under high temperatures (Ahn et al., 2003; Liu et al., 2014). Ten engineered thermotolerant *S. cerevisiae* strains were constructed by transformation with heat-resistant devices with *Thermoanaerobacter tengcongensis* Hsp and ubiquitin coding genes (Liu et al., 2014). Among them, the strains overexpressing *T.te-Tte2469*, *T.te-Gros2,* and *T.te-IbpA* had increased cell concentration (above 25%) and considerably greater survival than the control cells under high temperatures. Above all, the strain overexpressing *Hsp T.te-Gros2* demonstrated a four-fold rise in cellular growth at 42°C for 72 h compared to the control cells (Gao et al., 2016). Interestingly, *Y. lipolytica* cells, overexpressing native *Hsp90,* had increased thermotolerance when cultivated at temperatures rising from 30°C to 35°C (Zhang et al., 2022). However, the genes from thermophiles have not been reported to increase the thermotolerance of *Y. lipolytica*.

The current investigation aims to develop a thermotolerant *Y. lipolytica* strain with enhanced erythritol production at 35°C. In that sense, potential thermotolerance genes with diverse functions from different thermal preference strains were screened. Based on these genes, we have studied the thermotolerance and the ability of *Y. lipolytica* cells*,* which were transformed with the selected genes, to withstand oxidative stress. In the meantime, we proved that these cells expressed and produced erythritol. Furthermore, based on these genes, the effects of these cells’ growth and oxidative stress tolerance were investigated. This work showcases a significant improvement in thermotolerance and erythritol production of *Y. lipolytica*, which would significantly reduce energy consumption and ultimately favour the industrial production of erythritol.

## 2 Materials and methods

### 2.1 Chemical compounds and biological substances

Erythritol, 2′,7′-dichlorodihydrofluorescein diacetate (H2DCFDA, DCFH-DA) and propidium iodide solution were obtained from Solarbio (Beijing, China). New England Biolabs supplied the restriction endonucleases. KOD OneTM Master Mix -Blue- and PrimeSTAR® HS DNA Polymerase for the polymerase chain reactions (PCR) were purchased from Toyobo (Japan) or TaKaRa.

### 2.2 Yeast strains, culture media, and conditions

The *E. coli* and *Y. lipolytica* strains were employed here and are displayed in Supplementary Table S1. The *E. coli* cells were cultured at 37°C in LB (Jira et al., 2018) for plasmid construction. *Y. lipolytica* cells were grown at 30°C in YPD (Albers and Larsson., 2009). Where necessary, media were complemented with ampicillin (100 mg/L), nourseothricin (800 mg/L) or agarose (20 g/L). The shake-flask cultures were grown at 30°C, 35°C and 220 rpm in 100 mL of volume, containing 4 mL of fermentation medium, as previously described (Wang et al., 2020). All shake flasks were weighed before being placed into the shaker for incubation and then supplemented by adding the exact weight of sterilized water every 24 h to keep the volume unchanged.

### 2.3 Creation of thermotolerant gene devices and strains

Functional genes were obtained *via* PCR using the genome of *S. cerevisiae* Y-581 as templates, and others were chemically synthesized by Genewiz Inc., Suzhou, China. These genes were cloned into the pLPX-002 plasmid to give a series of plasmids. The detailed procedures of lithium acetate transformation for constructing the engineered strains are described in Holkenbrink et al. (Holkenbrink et al., 2018). Affirmative clones were verified on YPD-nourseothricin petri dishes and established by PCR detection and DNA sequencing. Information about the used primers, plasmids and engineered strains is provided in Supplementary Table S1.

### 2.4 Determination of cell concentration

The growth characteristics of the strains were measured under fermentation conditions. Each strain was at a primary cell density of OD_600_ = 0.1 and was grown in 100 mL of shake flasks with 4 mL of fermentation media and then cultured for 72 h at 35 C and shaking at 220 rpm. The UV-1800 spectrophotometer (Shimadzu, Kyoto, Japan) was used for cell growth optical density measurements (OD_600_).

### 2.5 Measurements of oxidative stress tolerance

The ability of the cells to tolerant oxidative stress was done following the protocol by Konzock et al. (Konzock and Norbeck., 2020). H_2_O_2_ was applied for oxidative stress induction at 50 mM final concentration. The cells were successively diluted in 1/10 steps. Cellular drops of 5 μL were grown on YPD-nourseothricin petri dishes at 30°C. At the same time, the cells were dissolved in PBS and 5 μg/mL PI (1 mg/mL stock in water). Cells were incubated for 20 min in the dark at room temperature (Kwolek-Mirek and Zadrag., 2014)^,^ and the cell viability based on the fluorescent dye was measured by flow cytometry (BD FACSAria III) (Qiu et al., 2021). The ROS concentration was measured following previously established procedures (Xu et al., 2017). Cells were collected and diluted to 0.3 OD_600_. They were mixed with 5 nmol of DCFH-DA for each sample and incubated at 37°C for an hour. Fluorescence was measured at 495 nm.

### 2.6 Analytical methods for erythritol and glucose measurements

Erythritol and glucose were detected by HPLC (Prominence UFLC, Shimadzu Corporation). The mobile phase was 5 μM sulfuric acid solution on an organic acid column (Aminex HPX-87H Ion Exclusion Column 300 mm × 7.8 mm; Catalog 125-0140; Bio-Rad Laboratories, Inc., United States). The entire protocol was published previously (Wang et al., 2020).

## 3 Results and discussion

### 3.1 Selection of heat-resistant genes

Improving the heat resistance of yeast strains is of great significance for reducing the energy cost required for cooling bioreactors in industrial production processes. However, thermotolerance is a complex phenotype involving various aspects of cellular physiology and metabolism (Alper et al., 2006; Liu et al., 2021). Large amounts of reactive oxygen species (ROS) are generated under higher temperatures, causing severe oxidative damage to cells, such as lipid peroxidation in cellular membranes, nucleic acid damage and increased toxicity by oxidation of proteins (Ahn et al., 2003). Several molecules have been reported to play essential roles against heat-induced oxidative stress in *S. cerevisiae* (Morano et al., 2012). Furthermore, recent data show that proteins synthesized by heat-inducible genes from different thermotolerant species, also designated as thermophiles, enhance heat resistance and thermotolerance in yeast species (Liu et al., 2014; Luan et al., 2014; Wang et al., 2015).

The high content of specific amino acids, such as Ile, Pro, Glu, Arg, and lower content of amino acids such as Cys, Ser, Thr, Asn and Asp make the heat-inducible proteins to natural proteins and valuable biological components for improving the thermotolerance of yeast (Ahn et al., 2003; Liu et al., 2014; Luan et al., 2014; Wang et al., 2015). In addition, trehalose accumulation and increased energy supply by affecting some heat-inducing genes in yeast itself (such as *TPS1* and *CDC19*) can help the strain resist heat damage (Gao et al., 2016). Furthermore, overexpression of the heat-inducing genes may contribute to maintaining cell wall integrity (Liu et al., 2014). Therefore, to improve the thermotolerance of *Y. lipolytica*, we screened and classified thirty potential heat-inducible genes which coded for stress-responsive proteins such as the Hsp family, ATPases, ubiquitin ligases, antioxidant enzymes and nucleic acid protectors from *S. cerevisiae* and different thermophilic strains such as *Thermus thermophilus* HB8, *Thermoanaerobacter tengcongensis* MB4, *Pyrococcus furiosus* and others. Information about all these genes was provided in Table 1; Supplemenetary Table S2. These stress-related genes were obtained by PCR or gene synthesis.

**TABLE 1:** Information of the functional genes used in this study.

Categories	Functions	Genes	Sources
Stress response proteins	Proteins that can protect against heat-induced oxidative and environmental stress.	Hsf1 (SC), Skn7, Msn2 (SC), Msn4	*Saccharomyces cerevisiae*
		Msn2 (KM), Hsf1 (KM)	*Kluyveromyces marxianus*
Heat shock proteins	Proteins that can protect thermally damaged proteins and improve the thermal stability of soluble proteins, SOD and proton pumps in stressed cells.	Hsp104, Hsp12	*Saccharomyces cerevisiae*
		Hsp10	*Thermus thermophilus* HB8
		SHsp	*Pyrococcus furiosus*
		Sso2427	*Sulfolobus solfatara*
		Gros2, Ibpa, Groes	*Thermoanaerobacter tengcongensis* MB4
ATPases	ATPase can transport hydrogen against the concentration gradient through membrane-integrated glycoproteins and stabilize the intracellular pH.	Pma1, Vma2, Vma3	*Saccharomyces cerevisiae*
Ubiquitin ligases	Ubiquitin ligases play a significant role in protecting cells against acute proteotoxic stress.	Hul5, Hrd1, Rma1, San1	*Saccharomyces cerevisiae*
		Tte2469	*Thermoanaerobacter tengcongensis* MB4
Antioxidant enzymes	Antioxidant enzymes are the primary protectors against oxidative damage.	Sod1, Cta1, Ctt1, Tsa1, Ahp1, Prx1	*Saccharomyces cerevisiae*
		Bifunctional glutathione synthase gene (GCSGC)	*Streptococcus thermophilus*
Nucleic acid protectors	Nucleic acid protectors are helpful for transcriptional regulation and the establishment of new protein homeostasis.	Cspl	*Bacillus coagulans* 2–6

### 3.2 Screening of effective heat-resistant gene devices

**FIGURE 1:**
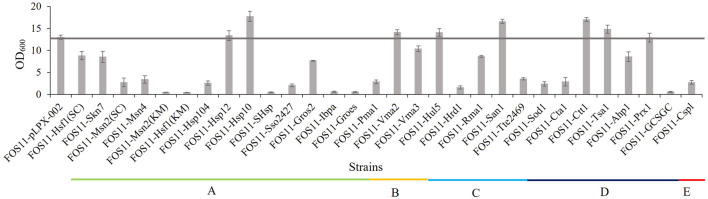
The cell density of engineered and control strains after 72 h cultured at 35°C. **(A).** Engineered strains transformed with plasmids containing different heat shock protein genes. **(B)**. Engineered strains transformed with plasmids containing different ATPase genes. **(C)**. Engineered strains transformed with plasmids containing different ubiquitin ligase genes. **(D)**. Engineered strains transformed with plasmids containing different antioxidant enzyme genes. **(E)**. Engineered strains transformed with plasmids containing different nucleic acid protector genes.

Considering the possible metabolic burden on cell growth by protein expression, we selected the medium-strength promoter *EXP1p* and the low-copy plasmid pLPX-002 to construct the heat-resistant modules. The resulting plasmids were transformed into *Y. lipolytica* strain FOS11 and allowed the generation of different engineered strains for preliminary screening by detecting the optical cell density at 35°C. As shown in Figure 1, the engineered strains FOS11-Hsp12, FOS11-Vma2, FOS11-Hul5, FOS11-San1, FOS11-Tsa1, FOS11-Prx1, FOS11-Hsp10 and FOS11-Ctt1 showed better growth compared with the control strain FOS11-pLPX-002. Among them, the OD_600_ values of FOS11-San1, FOS11-Hsp10 and FOS11-Ctt1 showed 27.71%, 36.49% and 30.95% increase compared with the control strain, respectively. Except these eight modules showed a positive effect on cell growth, the rest twenty-two constructs led to worse cell growth, even almost lethality compared with the control. Still, these results suggested that introducing heat-resistant devices could improve the thermotolerance of *Y. lipolytica*, especially in the yeast transformants with *San1*, *Hsp10* and *Ctt1*.

*Hsp10 (GroES)* was obtained from *Thermus thermophilus,* demonstrating optimum growth at 80°C (Laksanalamai et al., 2004). Studies have shown that GroES was used firstly for structural analyses among all heat shock proteins. Then, it formed a GroESL system with GroEL (also called Hsp60, which interacted with at least approximately 250 different cytosolic protein), which potentiated the proteins in non-native conformations to fold correctly and play an essential role in improving cell heat resistance (Vabulas et al., 2010). Protein homeostasis is finely regulated by protein quality control, which allows the removal of misfolded or impaired proteins from the cells. The process is mainly driven by the ubiquitin-proteasome system in eukaryotic cells (Ibarra et al., 2016). San1, one of the ubiquitin ligases, interacts directly with mismatched substrates in the nucleus and eliminates them (Rosenbaum et al., 2011). Heat shock induces significant oxidative stress, inducing the expression of the catalase enzymes as the first and most potent response. It was reported that the cytosolic catalase T, coded by *Ctt1*, protects cells from oxidative damage caused by hydrogen peroxide (Hiltunen et al., 2003; Martins et al., 2019). The expression of *Ctt1* may help cells clear away large amounts of ROS produced at high temperatures. Taken together, these results might be why the expression of *Hsp10*, *San1* and *Ctt1* enhanced the growth of *Y. lipolytica* at 35°C.

Proteins with different functions could improve the growth of *Y. lipolytica* at 35°C, suggesting that high-temperature conditions could induce different physiological and metabolic stress. However, in our studies, proteins with the same function resulted in little improvement or even worse growth. The possible reason may be due to the properties of the proteins themselves, such as enzyme activity and protein expression level, and the specific mechanisms involved in stress tolerance were not compatible with the growth and metabolism of host strains.

### 3.3 Antioxidant analysis of thermo-tolerant engineered strains

**FIGURE 2:**
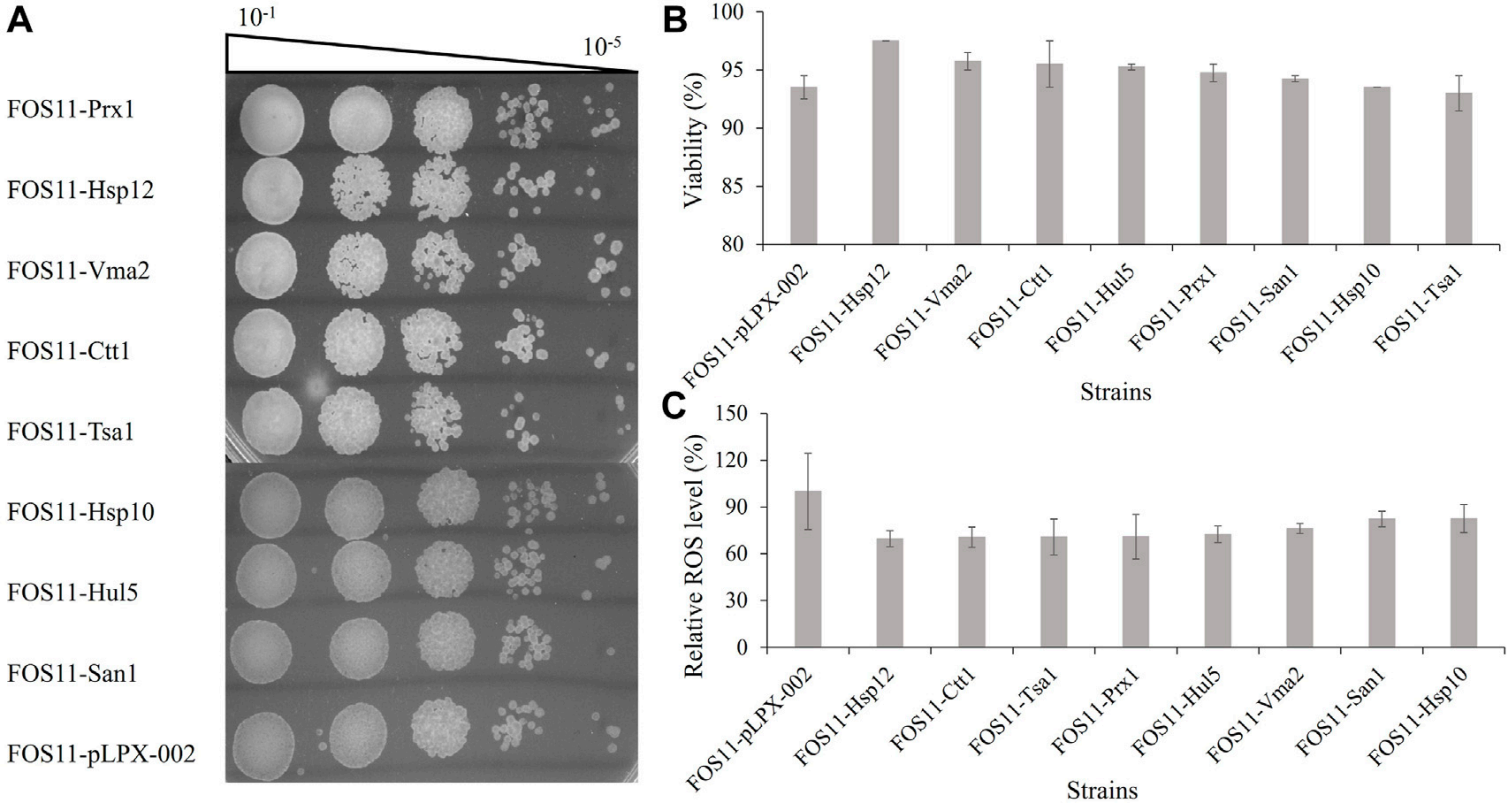
Characterization of the engineered strains treated with 50 mM H_2_O_2_. **(A)**. Investigation of colony forming units of the engineered and control strains by spotted plate method. **(B)**. Detection of cell viability based on the fluorescent dye of engineered and control strains by flow cytometry. **(C)**. Measurement of the intracellular ROS levels of engineered and control strains based on the fluorescent by microplate reader.

High temperature makes yeast cells accumulate a large amount of ROS, causing damage to cell growth and product biosynthesis (Gómez-Pastor et al., 2010). To further test the antioxidative effect of the eight effective heat-resistant transformant strains, we performed antioxidant analysis on engineered strains during the exponential phase (Supplementary Figure S2) using hydrogen peroxide (H_2_O_2_) to generate the ROS through the spotting test. As shown in Figure 2A, there was little difference between the eight engineered strains and the control. Therefore, introducing these heat-resistant devices did not alter the reproductive capacity of the engineered strains.

Colourimetric or fluorescent methods for measuring cell viability are faster and more correct than the standard approaches as they simultaneously measure cells that can and cannot reproduce but are still viable (Kwolek-Mirek and Zadrag., 2014). Staining cells with methylene blue or propidium iodide (PI) is widely used. PI enters only diseased cells and allows assessment of cellular viability on a single-cell level. Figure 2B demonstrated the augmented cellular viability of the eight engineered strains, especially FOS11-Hsp12, in which the cell viability was improved to 97.5%, which was increased by 4.3% compared with the control strain FOS11-pLPX-002 (93.5%). Welker et al. explored the structure and heat-resistant mechanism of Hsp12 and showed that it was fully unfolded and existed as a soluble cytoplasmic protein under normal conditions. However, under heat shock conditions, it interacted with negatively charged lipids to form helical structure and inserted into the cell membrane to increase its stability, thus protecting the cells from heat damage (Welker et al., 2010). Our data showed that though the expression of *Hsp12* does not improve the engineered strain’s reproductive capacity, it improved its cell viability under stress conditions. In yeast cells, negatively charged lipids, such as phospholipid inositol, are the main components of the membrane surface, accounting for about 20%–25% of the total lipid content (Welker et al., 2010). Therefore, the expression of *Hsp12* may stabilize cell membranes by regulating cell membrane fluidity at 35°C.

DCFH-DA is a commonly applied fluorometric probe for measuring oxidative stress. DCFH-DA penetrates the cells and is hydrolyzed to the DCFH carboxylate anion, thus forming dichlorofluorescein (DCF), which has fluorescence relevant to oxidation levels in the cells (Yılancıoğlu et al., 2014). To confirm whether these heat-resistant gene vehicles improved the antioxidant capacity of the studied strain, we determined ROS production by DCFH-DA. As shown in Figure 2C, the relative ROS level of all the engineered strains was considerably lesser than in control. The relative ROS level was 27.47%–30.34% lower than in the control strain FOS11-pLPX-002. The overexpression of the eight heat-resistant genes reduced the ROS levels of the strains. It made *Y. lipolytica* cells resistant to oxidative damage, which might be why the engineered strains acquired thermotolerance.

### 3.4 Enhanced erythritol production efficiency in thermotolerant-engineered strains

Erythritol is broadly applied in food and pharmaceuticals productions as it does not stimulate insulin production and produces fewer calories than conventional sugar (Carly et al., 2017; Janek et al., 2017). Several research teams have achieved great success in engineering *Y. lipolytica* strains capable of efficiently producing sugar alcohols (Mirończuk et al., 2017; Cheng et al., 2018; Mirończuk et al., 2019; Fickers et al., 2020). However, these yeast strains grew best at relatively low temperatures of 28°C–30°C (Gemperlein et al., 2019). Higher fermentation temperatures endangered them by inhibiting the functions of enzymes involved in PPP, which is responsible for erythritol synthesis (Fang et al., 2011). Consequently, increasing the thermotolerance of *Y. lipolytica,* even with several degrees, is of enormous advantage for effective erythritol production.

**FIGURE 3:**
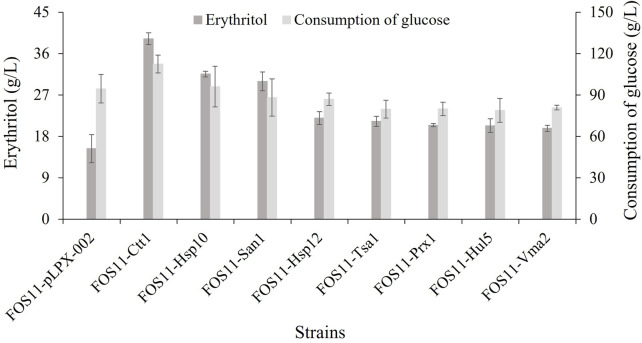
The production of erythritol and the consumption of glucose of control and engineered strains at 35°C. Data represent the mean ± SD of three biological replicates.

The above-discussed results showed that the heat resistance of the eight engineered strains had been improved. Because of this, we studied the erythritol production effectiveness of these engineered strains at 35°C. As shown in Figure 3; Table 2, these transformed strains had increased erythritol production compared to the control strain FOS11-pLPX-002. Significantly, the erythritol titer, yield and productivity of the strain FOS11-Ctt1 were improved to 39.25 g/L, 0.348 g/g glucose, and 0.55 g/L/h. This increased by 156%, 86% and 161%, respectively. For FOS11-San1, these parameters were equal to 29.95 g/L, 0.307 g/g glucose, and 0.42 g/L h, which was an estimated increase of 95%, 64%, and 100%, respectively. For FOS11-Hsp10, we had 31.58 g/L, 0.338 g/g glucose, and 0.44 g/L h, which increased by 106%, 81%, and 110%, respectively.

**TABLE 2:** Summary of fermentation profiles of engineered and control strains at 35°C.

Strains	Erythritol (g/L)	Q_ery_ (g/L/h)	Y_ery_ (g/g glucose)	OD_600_
FOS11-pLPX-002	15.33 ± 3.05	0.21 ± 0.042	0.187 ± 0.02	12.99 ± 0.47
FOS11-Hsp12	22 ± 1.4	0.31 ± 0.019	0.253 ± 0.017	13.37 ± 0.13
FOS11-Hsp10	31.58 ± 0.62	0.44 ± 0.009	0.338 ± 0.064	17.73 ± 1.17
FOS11-Hul5	20.32 ± 1.48	0.28 ± 0.02	0.233 ± 0.03	14.07 ± 0.87
FOS11-San1	29.95 ± 2.04	0.42 ± 0.028	0.307 ± 0.007	16.59 ± 0.46
FOS11-Vma2	19.75 ± 0.68	0.27 ± 0.01	0.244 ± 0.008	14.13 ± 0.55
FOS11-Ctt1	39.25 ± 4.6	0.55 ± 0.064	0.348 ± 0.026	17.01 ± 0.44
FOS11-Prx1	20.43 ± 0.34	0.28 ± 0.005	0.257 ± 0.016	12.9 ± 0.99
FOS11-Tsa1	21.26 ± 1.11	0.3 ± 0.015	0.269 ± 0.037	14.82 ± 0.91

**FIGURE 4:**
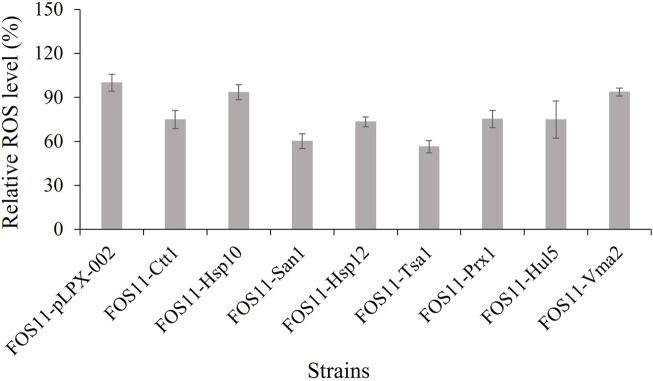
Comparison of the intracellular ROS level of engineered and control strains during fermentation at 35°C. Data represent the mean ± SD of three biological replicates.

We also compared the intracellular ROS levels of the engineered and control strains at 27 h of fermentation (exponential growth phase, Supplementary Figure S2). As shown in Figure 4, the strains’ relative ROS level was lower compared to the control strain. These results were consistent with previous assay data and suggested that the lower ROS level caused by the introduction of thermo-tolerant devices favoured the erythritol production of *Y. lipolytica*. We also evaluated the erythritol production capacity of the strains at 30°C. As shown in Supplementary Figure S1, there was little difference between the engineered strains and the control. This meant that the introduction of these heat-resistant devices had no effect on the erythritol production efficiency of the strain under average temperatures and further confirmed the acquired thermotolerance and enhanced erythritol production was the result of the expression of heat-resistant devices.

**FIGURE 5:**
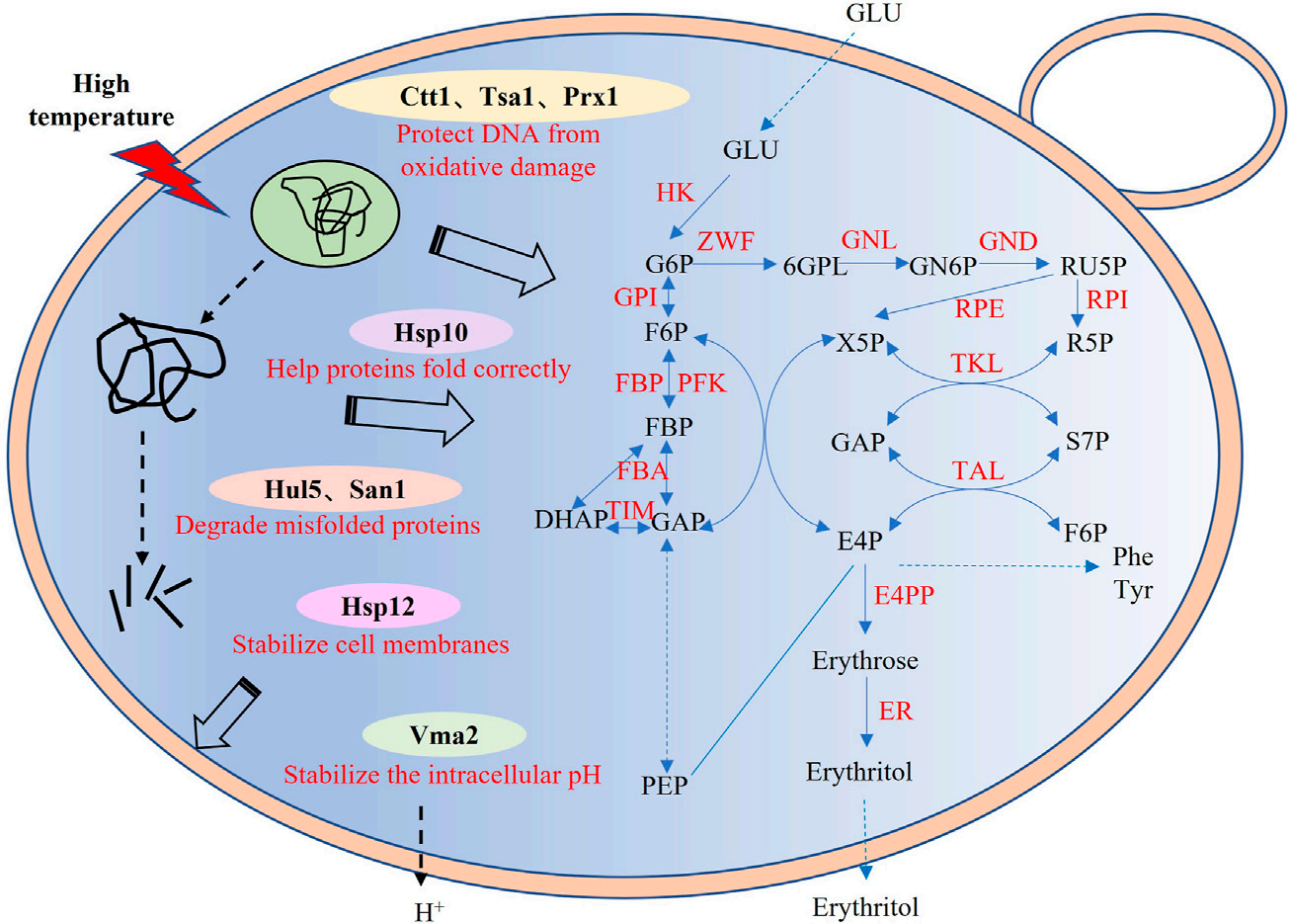
Synthetic pathway of erythritol and potential protective mechanism of heat-resistant devices. GLU, glucose; G6P, glucose-6-P; F6P, fructose-6-P; FBP, fructose-1,6-2P; DHAP, dihydroxyacetone phosphate; GAP, glyceraldehyde-P; Ru5P, ribulose-5P; X5P, xylulose-5-P; R5P, ribose-5-P; S7P, sedoheptulose-7-P; E4P, Erythrose-4-P; 6GPL, 6-P-gluconolactone; GN6P, 6-P-gluconate; PEP, phosphoenolpyruvate; HK, hexokinase; GPI, glucose-6-phosphate isomerase; FBP, fructose-1,6-bisphosphatase; PFK, 6-phosphofructokinase; RPE, ribulose-phosphate 3-epimerase; FBA, fructose-bisphosphate aldolase; TIM, triosephosphate isomerase; ZWF, glucose-6-phosphate 1-dehydrogenase; RPI, ribose 5-phosphate isomerase; GND, 6-phosphogluconate dehydrogenase; TKL, transketolase; TAL, transaldolase; GNL, 6-phosphogluconolactonase; E4PP, erythrose 4-phosphate phosphatase; XR, xylitol reductase.

Previous study has shown that Wang et al. improved the thermotolerance and the erythritol productivity of *Y. lipolytica* (Wang et al., 2020). However, they only tested the ubiquitin ligase Rsp5 from *S. cerevisiae* and the engineered strain only has an efficient production of erythritol at 33°C but not 35°C. In our study, we screened and tested thirty potential thermotolerant genes with diverse functions from different thermal preference strains. The results showed that eight proteins with different functions could improve the growth and erythritol production of *Y. lipolytica* at 35°C (Figure 5). It indicated that the co-expression of these heat-resistant genes may further improve the performance of *Y. lipolytica* under high temperature conditions, especially the three genes *Ctt1*, *San1* and *Hsp10*. In conclusion, a synthetic biology-based method was used to increase the thermotolerance of *Y. lipolytica* cells and their capacity to synthesize erythritol. This study provides a new heat-resistant device for improving the thermotolerance and erythritol biosynthetic efficiency of *Y. lipolytica*, laying the foundation for building a low-energy consumption cell factory for sugar alcohols production.

## Data Availability

The original contributions presented in the study are included in the article/Supplementary Material, further inquiries can be directed to the corresponding author.

## References

[B1] Abbasi A. R. Liu J. Wang Z. Zhao A. Ying H. Qu L. (2021). Recent advances in producing sugar alcohols and functional sugars by engineering *Yarrowia lipolytica* . Front. Bioeng. Biotechnol. 9, 648382. 10.3389/fbioe.2021.648382 33777917 PMC7992007

[B2] Ahn J. Jang H. W. Lee H. Choi E. Haam S. Oh T. K. (2003). Overexpression of thermoalkalophilic lipase from *Bacillus stearothermophilus* L1 in *Saccharomyces cerevisiae* . J. Microbiol. Biotechnol. 13, 451–456.

[B3] Albers E. Larsson C. (2009). A comparison of stress tolerance in YPD and industrial lignocellulose-based medium among industrial and laboratory yeast strains. J. Ind. Microbiol. Biotechnol. 36, 1085–1091. 10.1007/s10295-009-0592-1 19462190

[B4] Alper H. Moxley J. Nevoigt E. Fink G. R. Stephanopoulos G. (2006). Engineering yeast transcription machinery for improved ethanol tolerance and production. Sci 314, 1565–1568. 10.1126/science.1131969 17158319

[B5] Carly F. Vandermies M. Telek S. Steels S. Thomas S. Nicaud J. M. (2017). Enhancing erythritol productivity in *Yarrowia lipolytica* using metabolic engineering. Metab. Eng. 42, 19–24. 10.1016/j.ymben.2017.05.002 28545807

[B6] Cheng H. Wang S. Bilal M. Ge X. Zhang C. Fickers P. (2018). Identification, characterization of two NADPH-dependent erythrose reductases in the yeast *Yarrowia lipolytica* and improvement of erythritol productivity using metabolic engineering. Microb. Cell Fact. 17, 133. 10.1186/s12934-018-0982-z 30157840 PMC6114734

[B7] Costa D. A. de Souza C. J. Costa P. S. Rodrigues M. dos Santos A. F. Lopes M. R. (2014). Physiological characterization of thermotolerant yeast for cellulosic ethanol production. Appl. Microbiol. Biotechnol. 98, 3829–3840. 10.1007/s00253-014-5580-3 24535257 PMC3973951

[B8] Fang N. N. Ng A. H. Measday V. Mayor T. (2011). Hul5 HECT ubiquitin ligase plays a major role in the ubiquitylation and turnover of cytosolic misfolded proteins. Nat. Cell. Biol. 13, 1344–1352. 10.1038/ncb2343 21983566 PMC4961474

[B9] Fickers P. Cheng H. Sze Ki Lin C. (2020). Sugar alcohols and organic acids synthesis in *Yarrowia lipolytica*: Where are we? Microorganisms 8, 574. 10.3390/microorganisms8040574 32326622 PMC7232202

[B10] Gao L. Liu Y. Sun H. Li C. Zhao Z. Liu G. (2016). Advances in mechanisms and modifications for rendering yeast thermotolerance. J. Biosci. Bioeng. 121, 599–606. 10.1016/j.jbiosc.2015.11.002 26685013

[B11] Gemperlein K. Dietrich D. Kohlstedt M. Zipf G. Bernauer H. S. Wittmann C. (2019). Polyunsaturated fatty acid production by *Yarrowia lipolytica* employing designed myxobacterial PUFA synthases. Nat. Commun. 10, 4055. 10.1038/s41467-019-12025-8 31492836 PMC6731297

[B12] Gómez-Pastor R. Pérez-Torrado R. Cabiscol E. Ros J. Matallana E. (2010). Reduction of oxidative cellular damage by overexpression of the thioredoxin *TRX2* gene improves yield and quality of wine yeast dry active biomass. Microb. Cell Fact. 12, 9. 10.1186/1475-2859-9-9 PMC2835662 20152017

[B13] Hiltunen J. K. Mursula A. M. Rottensteiner H. Wierenga R. K. Kastaniotis A. J. Gurvitz A. (2003). The biochemistry of peroxisomal β-oxidation in the yeast *Saccharomyces cerevisiae* . FEMS Microbiol. 27, 35–64. 10.1016/S0168-6445(03)00017-2 12697341

[B14] Holkenbrink C. Dam M. I. Kildegaard K. R. Beder J. Dahlin J. Doménech Belda D. (2018). EasyCloneYALI: CRISPR/Cas9-eased synthetic toolbox for engineering of the yeast *Yarrowia lipolytica* . Biotechnol. J. 13, 1700543. 10.1002/biot.201700543 29377615

[B15] Ibarra R. Sandoval D. Fredrickson E. K. Gardner R. G. Kleiger G. (2016). The San1 ubiquitin ligase functions preferentially with ubiquitin-conjugating enzyme Ubc1 during protein quality control. J. Biol. Chem. 291, 18778–18790. 10.1074/jbc.M116.737619 27405755 PMC5009252

[B16] Janek T. Dobrowolski A. Biegalska A. Mirończuk A. M. (2017). Characterization of erythrose reductase from *Yarrowia lipolytica* and its influence on erythritol synthesis. Microb. Cell Fact. 16, 118. 10.1186/s12934-017-0733-6 28693571 PMC5504726

[B17] Jira J. Rezek B. Kriha V. Artemenko A. Matolínová I. Skakalova V. (2018). Inhibition of *E. coli* growth by nanodiamond and graphene oxide enhanced by Luria-Bertani medium. Nanomater. (Basel). 8, 140. 10.3390/nano8030140 PMC5869631 29494507

[B18] Konzock O. Norbeck J. (2020). Deletion of MHY1 abolishes hyphae formation in *Yarrowia lipolytica* without negative effects on stress tolerance. PloS One 15, 0231161. 10.1371/journal.pone.0231161 PMC7122783 32243483

[B19] Kwolek-Mirek M. Zadrag-Tecza R. (2014). Comparison of methods used for assessing the viability and vitality of yeast cells. FEMS yeast. Res. 14, 1068–1079. 10.1111/1567-1364.12202 25154541

[B20] Laksanalamai P. Whitehead T. A. Robb F. T. (2004). Minimal protein-folding systems in *hyperthermophilic archaea* . Nat. Rev. Microbiol. 2, 315–324. 10.1038/nrmicro866 15031730

[B21] Liu Y. Lin Y. Guo Y. Wu F. Zhang Y. Qi X. (2021). Stress tolerance enhancement via SPT15 base editing in *Saccharomyces cerevisiae* . Biotechnol. Biofuels. 14, 155. 10.1186/s13068-021-02005-w 34229745 PMC8259078

[B22] Liu Y. Zhang G. Sun H. Sun X. Jiang N. Rasool A. (2014). Enhanced pathway efficiency of *Saccharomyces cerevisiae* by introducing thermo-tolerant devices. Bioresour. Technol. 170, 38–44. 10.1016/j.biortech.2014.07.063 25118151

[B23] Luan G. Dong H. Zhang T. Lin Z. Zhang Y. Li Y. (2014). Engineering cellular robustness of microbes by introducing the GroESL chaperonins from extremophilic bacteria. J. Biotechnol. 178, 38–40. 10.1016/j.jbiotec.2014.03.010 24637367

[B24] Martins D. Nguyen D. English A. M. (2019). Ctt1 catalase activity potentiates antifungal azoles in the emerging opportunistic pathogen *Saccharomyces cerevisiae* . Sci. Rep. 9, 9185. 10.1038/s41598-019-45070-w 31235707 PMC6591360

[B25] Mason A. B. Allen K. E. Slayman C. W. (2014). C-terminal truncations of the *Saccharomyces cerevisiae* PMA1 H^+^-ATPase have major impacts on protein conformation, trafficking, quality control, and function. Eukaryot. Cell. 13, 43–52. 10.1128/EC.00201-13 24186948 PMC3910955

[B26] Mirończuk A. M. Biegalska A. Dobrowolski A. (2017). Functional overexpression of genes involved in erythritol synthesis in the yeast *Yarrowia lipolytica* . Biotechnol. Biofuels. 10, 77. 10.1186/s13068-017-0772-6 28352301 PMC5366165

[B27] Mirończuk A. M. Kosiorowska K. E. Biegalska A. Rakicka-Pustułka M. Szczepańczyk M. Dobrowolski A. (2019). Heterologous overexpression of bacterial hemoglobin VHb improves erythritol biosynthesis by yeast Yarrowia lipolytica. Microbial. Cell Factories 18, 1–8. 10.1186/s12934-019-1231-9 PMC6794898 31615519

[B28] Morano K. A. Grant C. M. Moye-Rowley W. S. (2012). The response to heat shock and oxidative stress in *Saccharomyces cerevisiae* . Genetics 190, 1157–1195. 10.1534/genetics.111.128033 22209905 PMC3316637

[B29] Qiu X. Gu Y. Du G. Zhang J. Xu P. Li J. (2021). Conferring thermotolerant phenotype to wild-type *Yarrowia lipolytica* improves cell growth and erythritol production. Biotechnol. Bioeng. 118, 3117–3127. 10.1002/bit.27835 34009652

[B30] Qiu X. Xu P. Zhao X. Du G. Zhang J. Li J. (2020). Combining genetically-encoded biosensors with high throughput strain screening to maximize erythritol production in *Yarrowia lipolytica* . Metab. Eng. 60, 66–76. 10.1016/j.ymben.2020.03.006 32224262

[B31] Rosenbaum J. C. Fredrickson E. K. Oeser M. L. Garrett-Engele C. M. Locke M. N. Richardson L. A. (2011). Disorder targets misorder in nuclear quality control degradation: A disordered ubiquitin ligase directly recognizes its misfolded substrates. Mol. Cell. 41, 93–106. 10.1016/j.molcel.2010.12.004 21211726 PMC3042722

[B32] Ross M. (2018). Erythritol market size, shares-global industry revenue by top key companies. Maharashtra: Report Hive Research.

[B33] Sanchez Y. Taulien J. Borkovich K. A. Lindquist S. (1992). Hsp104 is required for tolerance to many forms of stress. EMBO J. 11, 2357–2364. 10.1002/j.1460-2075.1992.tb05295.x 1600951 PMC556703

[B34] Schenk C. Schulz V. Rosch A. von Wallbrunn C. (2017). Less cooling energy in wine fermentation–A case study in mathematical modeling, simulation and optimization. Food. Bioprod. process. 103, 131–138. 10.1016/J.FBP.2017.04.001

[B35] Shahsavarani H. Sugiyama M. Kaneko Y. Chuenchit B. Harashima S. (2012). Superior thermotolerance of *Saccharomyces cerevisiae* for efficient bioethanol fermentation can be achieved by overexpression of RSP5 ubiquitin ligase. Biotechnol. Adv. 30, 1289–1300. 10.1016/j.biotechadv.2011.09.002 21930195

[B36] Vabulas R. M. Raychaudhuri S. Hayer-Hartl M. Hartl F. U. (2010). Protein folding in the cytoplasm and the heat shock response. Cold Spring Harb. Perspect. Biol. 2, 004390. 10.1101/cshperspect.a004390 PMC2982175 21123396

[B37] Wang N. Chi P. Zou Y. Xu Y. Xu S. Bilal M. (2020). Metabolic engineering of *Yarrowia lipolytica* for thermoresistance and enhanced erythritol productivity. Biotechnol. Biofuels. 13, 176. 10.1186/s13068-020-01815-8 33093870 PMC7576711

[B38] Wang Q. Cen Z. Zhao J. (2015). The survival mechanisms of thermophiles at high temperatures: An angle of omics. Physiol. (Bethesda) 30, 97–106. 10.1152/physiol.00066.2013 25729055

[B39] Welker S. Rudolph B. Frenzel E. Hagn F. Liebisch G. Schmitz G. (2010). Hsp12 is an intrinsically unstructured stress protein that folds upon membrane association and modulates membrane function. Mol. Cell. 39, 507–520. 10.1016/j.molcel.2010.08.001 20797624

[B40] Xu P. Qiao K. Stephanopoulos G. (2017). Engineering oxidative stress defense pathways to build a robust lipid production platform in *Yarrowia lipolytica* . Biotechnol. Bioeng. 114, 1521–1530. 10.1002/bit.26285 28295166

[B41] Yılancıoğlu K. Cokol M. Pastirmaci I. Erman B. Çetiner S. (2014). Oxidative stress is a mediator for increased lipid accumulation in a newly isolated *Dunaliella salina* strain. PloS One 9, e91957. 10.1371/journal.pone.0091957 24651514 PMC3961284

[B42] Zhang Y. Zhang X. Xu Y. Xu S. Bilal M. Cheng H. (2022). Engineering thermotolerant *Yarrowia lipolytica* for sustainable biosynthesis of mannitol and fructooligosaccharides. Biochem. Eng. J. 187, 108604. 10.1016/j.bej.2022.108604

