# Supplementary material for: Enhancing the thermotolerance and erythritol production of Yarrowia lipolytica by introducing heat-resistant devices
Source: Front Bioeng Biotechnol. 2023 Feb 9;11:1108653. doi: 10.3389/fbioe.2023.1108653 (PMC9947466; doi:10.3389/fbioe.2023.1108653)
Supplement: Supplementary file 1 [file DataSheet1.docx]

Supplementary Material

**Table S1** Strains, gene cassettes, and primers used in this study.

| Strains/plasmids/primers | Genotype/sequences (5’-3’) | References |
| --- | --- | --- |
| ***E. coli* strain** |  |  |
| *E. coli* DH5α | Δ*(lacZYA-argF)* U169*,recA*1*,endA*1*, phoA, supE*44*, thi*-1*, relA*1 | Takara |
| ***Y. lipolylica* strains** |  |  |
| FOS11 | wild type | This lab |
| FOS11-Hsp10 | FOS11 pLPX-002-Hsp10 | This work |
| FOS11-Hsp104 | FOS11 pLPX-002-Hsp104 | This work |
| FOS11-Hsp12 | FOS11 pLPX-002-Hsp12 | This work |
| FOS11-SHsp | FOS11 pLPX-002-SHsp | This work |
| FOS11-Sso2427 | FOS11 pLPX-002-Sso2427 | This work |
| FOS11-gros2 | FOS11 pLPX-002- gros2 | This work |
| FOS11-Ibpa | FOS11 pLPX-002- Ibpa | This work |
| FOS11-Groes | FOS11 pLPX-002- Groes | This work |
| FOS11-Hsf1 (SC) | FOS11 pLPX-002- Hsf1 | This work |
| FOS11-Skn7 | FOS11 pLPX-002- Skn7 | This work |
| FOS11-Msn2 (SC) | FOS11 pLPX-002- Msn2 | This work |
| FOS11- Msn4 | FOS11 pLPX-002- Msn4 | This work |
| FOS11-Msn2 (KM) | FOS11 pLPX-002-Msn2 (KM) | This work |
| FOS11-Hsf1 (KM) | FOS11 pLPX-002-Hsf1 (KM) | This work |
| FOS11-Pma1 | FOS11 pLPX-002-Pma1 | This work |
| FOS11-Vma2 | FOS11 pLPX-002-Vma2 | This work |
| FOS11-Vma3 | FOS11 pLPX-002-Vma3 | This work |
| FOS11-Hul5 | FOS11 pLPX-002-Hul5 | This work |
| FOS11-Hrd1 | FOS11 pLPX-002-Hrd1 | This work |
| FOS11-Rma1 | FOS11 pLPX-002-Rma1 | This work |
| FOS11-San1 | FOS11 pLPX-002-San1 | This work |
| FOS11-Tte2469 | FOS11 pLPX-002-Tte2469 | This work |
| FOS11-Sod1 | FOS11 pLPX-002-Sod1 | This work |
| FOS11-Cta1 | FOS11 pLPX-002-Cta1 | This work |
| FOS11-Ctt1 | FOS11 pLPX-002-Ctt1 | This work |
| FOS11-Tsa1 | FOS11 pLPX-002-Tsa1 | This work |
| FOS11-Ahp1 | FOS11 pLPX-002-Ahp1 | This work |
| FOS11-Prx1 | FOS11 pLPX-002-Prx1 | This work |
| FOS11-GCSGC | FOS11 pLPX-002-GCSGC | This work |
| FOS11-Csp1 | FOS11 pLPX-002-Csp1 | This work |
| **Plasmids** |  |  |
| pCfB3405 | pORI1001-Nat-CEN1-USER | 16 |
| pLPX-001 | pCfB3405 derivative containing pXPR2-Nat | This lab |
| pLPX-002 | pLPX-001 derivative containing up intE_1-pEXP1-TT-dw intE_1 | This lab |
| pLPX-002-Hsp10 | pLPX-002 derivative containing *Hsp10* | This work |
| pLPX-002-Hsp104 | pLPX-002 derivative containing *Hsp104* | This work |
| pLPX-002-Hsp12 | pLPX-002 derivative containing *Hsp12* | This work |
| pLPX-002-SHsp | pLPX-002 derivative containing S*Hsp* | This work |
| pLPX-002-Sso2427 | pLPX-002 derivative containing *Sso2427* | This work |
| pLPX-002-Gros2 | pLPX-002 derivative containing *Gros2* | This work |
| pLPX-002-Ibpa | pLPX-002 derivative containing *Ibpa* | This work |
| pLPX-002-Groes | pLPX-002 derivative containing *Groes* | This work |
| pLPX-002-Hsf1 (SC) | pLPX-002 derivative containing *Hsf1* (SC) | This work |
| pLPX-002-Skn7 | pLPX-002 derivative containing *Skn7* | This work |
| pLPX-002-Msn2 (SC) | pLPX-002 derivative containing *Msn2* (SC) | This work |
| pLPX-002- Msn4 | pLPX-002 derivative containing *Msn4* | This work |
| pLPX-002-Msn2 (KM) | pLPX-002 derivative containing *Msn2* (KM) | This work |
| pLPX-002-Hsf1 (KM) | pLPX-002 derivative containing *Hsf1* (KM) | This work |
| pLPX-002-Pma1 | pLPX-002 derivative containing *Pma1* | This work |
| pLPX-002-Vma2 | pLPX-002 derivative containing *Vma2* | This work |
| pLPX-002- Vma3 | pLPX-002 derivative containing *Vma3* | This work |
| pLPX-002-Hul5 | pLPX-002 derivative containing *Hul5* | This work |
| pLPX-002-Hrd1 | pLPX-002 derivative containing *Hrd1* | This work |
| pLPX-002-Rma1 | pLPX-002 derivative containing *Rma1* | This work |
| pLPX-002-San1 | pLPX-002 derivative containing *San1* | This work |
| pLPX-002-Tte2469 | pLPX-002 derivative containing *Tte2469* | This work |
| pLPX-002-Sod1 | pLPX-002 derivative containing *Sod1* | This work |
| pLPX-002-Cta1 | pLPX-002 derivative containing *Cta1* | This work |
| pLPX-002-Ctt1 | pLPX-002 derivative containing *Ctt1* | This work |
| pLPX-002-Tsa1 | pLPX-002 derivative containing *Tsa1* | This work |
| pLPX-002-Ahp1 | pLPX-002 derivative containing *Ahp1* | This work |
| pLPX-002-Prx1 | pLPX-002 derivative containing *Prx1* | This work |
| pLPX-002-GCSGC | pLPX-002 derivative containing *GCSGC* | This work |
| pLPX-002-Csp1 | pLPX-002 derivative containing *Csp1* | This work |
| **Primers** | sequences (5’-3’) |  |
| Ahp1-F | GACATATCTACAGCAATGTCTGACTTAGTTAACAA | This work |
| Ahp1-R | ACCGGCAACGTGGGGCTACAAATGAGCCAAGACAC | This work |
| Cta1-F | GACATATCTACAGCAATGTCGAAATTGGGACAAGA | This work |
| Cta1-R | ACCGGCAACGTGGGGTCAAAATTTGGAGTTACTCG | This work |
| Ctt1-F | GACATATCTACAGCAATGCCAATAAGATCAATCAG | This work |
| Ctt1-R | ACCGGCAACGTGGGGTTAATTGGCACTTGCAATGG | This work |
| Hrd1-F | GACATATCTACAGCAATGGTGCCAGAAAATAGAAG | This work |
| Hrd1-R | ACCGGCAACGTGGGGCTAGATATGCTGGATAAATT | This work |
| Hsf1 (SC)-F | GACATATCTACAGCAATGTTAAACGATGATTCTAA | This work |
| Hsf1 (SC)-R | ACCGGCAACGTGGGGCTATTTCTTAGCTCGTTTGG | This work |
| Hsp12-F | GACATATCTACAGCAATGTCTGACGCAGGTAGAAA | This work |
| Hsp12-R | ACCGGCAACGTGGGGTTACTTCTTGGTTGGGTCTT | This work |
| Hsp104-F | GACATATCTACAGCAATGAACGACCAAACGCAATT | This work |
| Hsp104-R | ACCGGCAACGTGGGGTTAATCTAGGTCATCATCAA | This work |
| Hul5-F | GACATATCTACAGCAATGTTAAACTTCACCGGTCA | This work |
| Hul5-R | ACCGGCAACGTGGGGTTATGATAAGTCAAACCTGG | This work |
| Msn2 (SC)-F | GACATATCTACAGCAATGACGGTCGACCATGATTT | This work |
| Msn2 (SC)-R | ACCGGCAACGTGGGGTTAAATGTCTCCATGTTTTT | This work |
| Msn4-F | GACATATCTACAGCAATGCTAGTCTTCGGACCTAA | This work |
| Msn4-R | ACCGGCAACGTGGGGTCAAAAATCACCGTGCTTTT | This work |
| Pma1-F | GACATATCTACAGCAATGACTGATACATCATCCTC | This work |
| Pma1-R | ACCGGCAACGTGGGGTTAGGTTTCCTTTTCGTGTT | This work |
| Prx1-F | GACATATCTACAGCAATGTTTAGTAGAATTTGTAG | This work |
| Prx1-R | ACCGGCAACGTGGGGTTATTTCGACTTGGTGAATC | This work |
| Rma1-F | GACATATCTACAGCAATGGATGATATAAGCGGAAG | This work |
| Rma1-R | ACCGGCAACGTGGGGCTATACTGGCAAGTGACAGT | This work |
| San1-F | GACATATCTACAGCAATGAGTGAAAGTGGTCAAGA | This work |
| San1-R | ACCGGCAACGTGGGGTTATTGTGATGATCGTTGCT | This work |
| Skn7-F | GACATATCTACAGCAATGAGCTTTTCCACCATAAA | This work |
| Skn7-R | ACCGGCAACGTGGGGTTATGATAGCTGGTTTTCTT | This work |
| Sod1-F | GACATATCTACAGCAATGGTTCAAGCAGTCGCAGT | This work |
| Sod1-R | ACCGGCAACGTGGGGTTAGTTGGTTAGACCAATGA | This work |
| Tsa1-F | GACATATCTACAGCAATGGTCGCTCAAGTTCAAAA | This work |
| Tsa1-R | ACCGGCAACGTGGGGTTATTTGTTGGCAGCTTCGA | This work |
| Vma2-F | GACATATCTACAGCAATGGTTTTGTCTGATAAGGA | This work |
| Vma2-R | ACCGGCAACGTGGGGTTAGATTAGAGATTCTTCTT | This work |
| Vma3-F | GACATATCTACAGCAATGACTGAATTGTGTCCTGT | This work |
| Vma3-R | ACCGGCAACGTGGGGTTAACAGACAACATCTTGAG | This work |

**Table S2** Sequences of genes.

| Genes | Sequences |
| --- | --- |
| Ahp1 | ATGTCTGACTTAGTTAACAAGAAATTCCCAGCTGGCGACTACAAATTCCAATACATTGCTATCAGCCAAAGTGATGCTGACAGTGAATCTTGTAAGATGCCACAAACAGTTGAATGGTCCAAATTAATTTCTGAAAACAAGAAGGTTATCATTACCGGTGCTCCAGCTGCTTTCTCCCCAACCTGTACTGTCAGCCATATTCCAGGTTACATCAACTACTTGGATGAATTAGTTAAGGAAAAGGAAGTTGACCAAGTGATCGTTGTTACTGTTGACAACCCGTTCGCTAACCAAGCGTGGGCTAAGAGTTTAGGTGTTAAGGACACCACACACATCAAGTTTGCCTCCGACCCAGGCTGTGCTTTCACCAAATCCATTGGTTTCGAATTAGCCGTCGGTGACGGTGTTTACTGGAGTGGTAGATGGGCCATGGTTGTTGAAAACGGTATCGTTACTTACGCTGCCAAGGAAACCAACCCAGGTACCGATGTGACCGTTTCCTCAGTCGAAAGTGTCTTGGCTCATTTGTAG |
| Cta1 | ATGTCGAAATTGGGACAAGAAAAAAATGAAGTAAATTACTCTGATGTAAGAGAGGATAGAGTTGTGACAAACTCCACTGGTAATCCAATCAATGAACCATTTGTCACCCAACGTATTGGGGAACATGGCCCTTTGCTTTTGCAAGATTATAACTTAATTGATTCTTTGGCTCATTTCAACAGGGAAAATATTCCTCAAAGGAATCCACATGCTCATGGTTCTGGTGCCTTCGGCTATTTTGAAGTAACCGATGACATTACTGATATCTGCGGGTCTGCTATGTTTAGTAAAATTGGGAAAAGAACGAAATGTCTAACAAGATTTTCGACTGTGGGTGGTGATAAAGGTAGTGCCGACACGGTTCGTGATCCAAGGGGGTTTGCCACCAAATTCTACACTGAAGAAGGTAATTTAGATTGGGTCTACAATAATACACCGGTATTCTTTATCAGAGACCCTTCCAAGTTCCCTCACTTTATCCACACACAGAAGAGAAACCCACAAACCAACCTAAGGGATGCTGACATGTTTTGGGATTTCCTCACCACTCCTGAAAATCAGGTGGCCATTCATCAAGTAATGATCCTTTTTTCAGACCGTGGTACCCCTGCCAACTACCGTAGTATGCATGGTTATTCTGGTCATACCTATAAATGGTCCAATAAAAACGGAGATTGGCATTATGTGCAAGTTCATATCAAAACCGATCAAGGAATAAAGAATTTGACCATAGAAGAGGCTACCAAAATTGCGGGATCCAATCCAGATTACTGCCAGCAGGATTTATTTGAGGCTATTCAGAATGGAAACTATCCTTCCTGGACAGTTTATATTCAAACAATGACCGAACGCGATGCCAAAAAATTACCATTTTCAGTCTTTGATTTGACTAAAGTATGGCCTCAGGGGCAATTCCCTTTACGGCGTGTGGGTAAGATTGTTTTGAACGAGAATCCACTGAACTTCTTCGCACAGGTGGAACAAGCTGCCTTCGCCCCCAGTACCACGGTTCCTTACCAAGAAGCAAGCGCTGATCCAGTATTACAGGCCCGTTTGTTTTCATATGCGGATGCTCATAGATACAGGCTAGGTCCTAACTTCCATCAAATACCCGTAAACTGTCCATATGCATCTAAATTTTTCAATCCCGCTATCAGAGATGGACCGATGAATGTTAACGGCAACTTCGGCTCAGAACCTACATATTTGGCCAACGATAAATCGTACACGTATATCCAACAGGACAGACCCATTCAACAACACCAAGAGGTATGGAATGGGCCAGCTATCCCTTATCATTGGGCAACATCCCCAGGTGATGTAGATTTCGTGCAAGCAAGAAATCTCTACCGCGTTTTGGGTAAACAACCTGGACAGCAAAAGAACTTGGCATATAACATCGGCATTCATGTAGAAGGCGCCTGTCCTCAAATACAGCAGCGCGTTTATGATATGTTTGCTCGTGTTGATAAGGGACTATCTGAGGCAATTAAAAAAGTAGCTGAGGCAAAACATGCTTCTGAGCTTTCGAGTAACTCCAAATTTTGA |
| Ctt1 | ATGCCAATAAGATCAATCAGCTCAGCTTCACAAATGAACGTGTTCGGTAAAAAAGAAGAAAAGCAAGAAAAAGTTTACTCTCTACAAAACGGTTTTCCGTACTCTCATCACCCATACGCTTCTCAATACTCAAGACCAGACGGCCCTATCTTACTGCAAGACTTCCATCTGCTGGAAAATATCGCAAGTTTCGATAGAGAAAGAGTTCCGGAGCGTGTAGTCCATGCCAAAGGTGGTGGTTGTAGACTGGAGTTCGAACTAACAGATTCTTTGAGTGATATTACATACGCCGCTCCATACCAGAATGTGGGTTACAAATGTCCTGGTCTTGTTCGTTTTTCCACCGTTGGTGGTGAAAGTGGTACACCAGACACTGCAAGAGACCCAAGAGGTGTTTCTTTTAAATTCTATACCGAGTGGGGGAACCATGACTGGGTCTTCAACAATACTCCCGTCTTCTTCCTCAGAGACGCTATTAAGTTTCCCGTATTTATTCATTCGCAAAAGAGAGACCCTCAGTCTCATCTGAATCAGTTTCAGGACACTACCATATACTGGGATTATCTAACATTGAATCCGGAATCAATCCATCAAATAACTTACATGTTTGGTGATAGAGGTACTCCTGCTTCGTGGGCTAGTATGAACGCGTACTCTGGTCATTCCTTCATCATGGTCAACAAAGAAGGTAAGGACACATATGTGCAATTCCACGTCTTGTCGGATACTGGTTTTGAAACCTTGACTGGAGATAAGGCTGCTGAACTGTCAGGCTCCCACCCTGATTATAATCAGGCAAAGCTGTTCACTCAATTGCAAAATGGCGAAAAGCCAAAATTTAACTGTTATGTGCAAACAATGACACCCGAACAAGCAACTAAGTTCAGGTATTCGGTAAATGACCTAACGAAAATATGGCCACACAAGGAATTCCCTTTGAGAAAATTTGGTACCATCACCCTAACGGAGAATGTTGACAATTATTTCCAAGAAATTGAACAAGTTGCATTCAGTCCAACGAACACTTGTATCCCAGGTATTAAGCCTTCTAATGATTCCGTTCTACAAGCCAGACTTTTCTCCTATCCAGACACTCAACGTCATAGATTGGGAGCCAACTATCAGCAATTGCCCGTCAACAGACCAAGAAACTTGGGATGTCCATACTCCAAAGGTGATTCCCAATACACTGCCGAACAGTGTCCATTTAAAGCAGTGAACTTCCAAAGGGACGGCCCAATGAGTTACTACAATTTCGGTCCTGAGCCAAATTATATTTCCAGTTTACCAAATCAAACTCTGAAATTCAAAAATGAAGACAACGACGAAGTATCTGATAAGTTCAAAGGGATAGTTCTTGACGAAGTAACAGAAGTTTCTGTGAGAAAACAGGAACAAGACCAAATCAGAAACGAGCATATTGTTGATGCCAAAATTAATCAATATTACTACGTTTATGGTATTAGTCCACTAGACTTCGAACAGCCAAGAGCTCTATATGAAAAGGTATACAACGATGAACAGAAGAAATTATTCGTTCATAACGTTGTTTGCCACGCTTGTAAGATCAAAGATCCTAAAGTCAAAAAGAGAGTTACGCAATACTTTGGTTTGCTAAACGAAGATTTGGGTAAAGTCATTGCAGAAGGCTTGGGAGTTCCTTGGGAACCTGTTGACCTTGAAGGTTATGCCAAGACTTGGTCCATTGCAAGTGCCAATTAA |
| Hsf1 (SC) | ATGTTAAACGATGATTCTAATACGAAACTTATACAGTGGGCGGAGGATGGAAAATCTTTTATTGTCACGAATAGGGAGGAATTTGTGCACCAAATTTTACCAAAATATTTTAAACATTCCAATTTCGCTTCCTTTGTAAGACAATTGAACATGTATGGATGGCATAAAGTTCAAGATGTCAAGTCAGGATCAATTCAAAGTAGTTCAGATGATAAGTGGCAATTTGAAAATGAAAACTTCATTAGAGGTAGAGAAGATTTGCTGGAAAAAATAATCAGGCAGAAAGGTTCCTCCAATAACCATAATAGCCCTAGTGGTAACGGTAATCCAGCGAATGGTAGCAACATCCCTCTGGACAATGCCGCAGGAAGTAATAATAGCAATAATAACATCAGTAGTAGTAATTCATTTTTTAACAATGGTCATTTATTGCAGGGTAAAACACTAAGATTAATGAACGAAGCGAATCTTGGAGATAAGAATGATGTCACCGCGATTTTGGGGGAATTAGAGCAAATAAAATATAACCAGATTGCAATTTCCAAAGATTTACTAAGAATAAACAAAGATAATGAGTTATTATGGCAAGAGAATATGATGGCCAGGGAAAGACATAGAACCCAACAGCAAGCCTTGGAAAAAATGTTCAGATTCTTGACATCTATAGTCCCACACTTAGATCCCAAAATGATTATGGACGGGCTGGGAGATCCGAAAGTTAATAATGAAAAGCTAAACAGTGCGAATAACATTGGGTTAAATCGCGACAACACAGGCACTATAGATGAACTAAAATCCAACGATTCTTTCATAAACGATGATCGTAATTCTTTCACCAATGCTACAACCAACGCCCGTAATAACATGAGTCCCAACAATGATGACAATAGTATTGACACCGCTAGCACTAATACCACCAACAGAAAGAAAAATATAGATGAAAACATCAAAAATAACAACGACATAATTAATGACATTATATTTAATACCAACCTTGCCAACAATCTCAGCAATTACAATTCCAACAATAATGCTGGCTCGCCAATAAGGCCCTATAAACAAAGATATCTTTTGAAAAATAGAGCCAATTCCTCGACATCGAGTGAGAATCCAAGCCTAACGCCCTTTGATATCGAATCTAATAATGACCGCAAAATTTCAGAAATTCCTTTTGATGACGAAGAAGAAGAAGAAACGGATTTTAGGCCTTTTACCTCGCGAGATCCTAATAACCAAACGAGTGAAAACACTTTTGATCCAAACAGATTTACGATGCTCTCTGATGATGATTTAAAAAAAGATTCTCATACCAATGACAATAAACACAACGAAAGTGATCTTTTTTGGGACAACGTACATAGAAATATAGACGAACAAGATGCAAGACTCCAGAACTTGGAAAATATGGTTCACATACTTTCTCCTGGATATCCTAATAAGTCGTTCAACAACAAAACTTCCTCGACAAACACTAATTCCAATATGGAAAGTGCTGTCAACGTTAATAGCCCTGGTTTCAACTTACAGGATTATTTAACTGGAGAGTCTAATTCCCCCAATTCTGTTCATTCTGTTCCCTCCAATGGCAGCGGCTCCACACCGTTGCCCATGCCAAATGATAATGACACCGAGCACGCAAGTACAAGTGTCAATCAAGGCGAAAATGGAAGCGGATTAACGCCCTTCCTCACGGTAGATGATCACACACTAAACGACAATAACACTAGTGAGGGAAGTACAAGGGTGTCCCCCGATATAAAGTTCAGCGCCACTGAAAACACTAAAGTGAGTGATAACCTGCCAAGCTTTAATGACCACAGTTATTCCACCCAGGCCGACACGGCGCCCGAGAACGCTAAGAAAAGATTTGTGGAGGAAATACCGGAACCGGCTATAGTCGAAATACAGGACCCGACAGAGTACAACGATCACCGCCTGCCCAAACGAGCTAAGAAATAG |
| Hsp12 | ATGTCTGACGCAGGTAGAAAAGGATTCGGTGAAAAAGCTTCTGAAGCTTTGAAGCCAGACTCTCAAAAGTCATACGCTGAACAAGGTAAGGAATACATCACTGACAAGGCCGACAAGGTCGCTGGTAAGGTTCAACCAGAAGACAACAAGGGTGTCTTCCAAGGTGTCCACGACTCTGCCGAAAAAGGCAAGGATAACGCTGAAGGTCAAGGTGAATCTTTGGCAGACCAAGCTAGAGATTACATGGGAGCCGCCAAGTCCAAGTTGAACGATGCCGTCGAATATGTTTCCGGTCGTGTCCACGGTGAAGAAGACCCAACCAAGAAGTAA |
| Hrd1 | ATGGTGCCAGAAAATAGAAGGAAACAGTTGGCAATTTTTGTAGTTGTCACATATTTGCTCACATTTTATTGCGTGTATTCAGCCACCAAGACAAGCGTTTCCTTTTTGCAAGTAACACTGAAGCTAAATGAAGGCTTCAATCTAATGGTTTTATCGATATTCATCTTATTAAATTCTACCTTACTATGGCAGCTCCTAACGAAACTATTATTTGGTGAACTGAGGCTTATTGAGCATGAGCACATTTTTGAAAGGTTACCATTTACCATTATAAACACCTTGTTTATGTCCTCACTGTTCCACGAGCGGTATTTTTTCACAGTGGCATTTTTTGGACTATTACTACTCTATCTGAAAGTTTTCCATTGGATTTTAAAGGATAGGCTGGAGGCCTTATTACAGTCAATAAATGATTCCACCACAATGAAAACCCTTATCTTTAGTAGATTCTCATTTAACCTCGTACTATTGGCGGTTGTAGACTACCAGATAATAACACGATGCATCTCCTCCATATATACGAACCAAAAGAGTGATATTGAATCCACATCCCTTTACCTGATACAAGTAATGGAGTTTACCATGCTTTTGATTGATTTGCTAAATTTATTCCTACAGACTTGTTTGAATTTCTGGGAATTTTATCGCTCACAACAAAGTCTGTCTAATGAGAACAACCATATTGTCCATGGCGATCCTACAGATGAAAACACGGTTGAGTCTGATCAATCTCAGCCAGTGCTGAATGACGACGACGATGACGACGATGATGATAGACAATTTACCGGCCTGGAGGGTAAATTCATGTATGAAAAAGCAATTGACGTATTCACAAGATTCTTAAAAACGGCACTTCATTTGTCTATGCTAATACCATTTAGGATGCCTATGATGCTTTTGAAAGATGTGGTGTGGGATATCTTGGCACTATATCAAAGTGGCACAAGTTTGTGGAAAATCTGGAGAAATAACAAACAGCTCGACGACACTCTTGTCACTGTCACCGTAGAACAGCTACAAAATTCTGCAAATGATGACAATATTTGTATCATTTGTATGGATGAGTTAATACATTCTCCAAACCAGCAGACGTGGAAGAATAAAAACAAGAAACCCAAAAGGTTACCTTGTGGCCACATACTTCATTTGTCGTGTTTAAAGAATTGGATGGAACGTTCTCAGACTTGTCCTATTTGTAGATTGCCTGTCTTTGATGAAAAAGGTAATGTTGTGCAAACGACTTTCACTTCCAATAGTGATATCACGACACAGACCACCGTAACAGATAGCACTGGGATAGCGACAGATCAACAAGGTTTCGCAAACGAAGTAGATCTACTTCCCACAAGAACAACTTCCCCTGATATAAGGATAGTGCCTACTCAAAATATAGACACATTAGCAATGAGAACAAGGTCAACCTCTACACCATCTCCTACGTGGTATACGTTCCCATTACATAAAACTGGTGATAATTCTGTTGGGTCAAGCCGATCAGCCTACGAATTTTTGATCACAAATTCAGATGAGAAAGAAAATGGTATTCCTGTCAAATTAACAATAGAAAATCACGAAGTAAATTCTCTGCATGGAGACGGGGGCGAGCAAATTGCCAAGAAAATTGTCATACCAGATAAATTTATCCAGCATATCTAG |
| Hsp104 | ATGAACGACCAAACGCAATTTACAGAAAGGGCTCTAACGATTTTGACGTTGGCTCAAAAATTGGCTTCGGATCATCAACATCCACAATTACAACCTATACATATTCTAGCTGCCTTCATTGAAACGCCAGAAGATGGATCAGTCCCTTACCTACAGAATCTAATTGAGAAGGGCCGTTACGACTATGATCTTTTCAAGAAAGTGGTTAATAGAAATCTAGTAAGAATTCCTCAACAGCAACCTGCACCTGCGGAGATAACTCCAAGTTATGCTTTGGGGAAAGTCCTTCAAGACGCTGCTAAGATTCAAAAACAACAGAAGGACTCATTTATAGCGCAAGACCATATATTGTTTGCTCTATTCAATGATTCGTCTATTCAGCAAATATTTAAGGAAGCTCAAGTAGATATTGAGGCCATCAAGCAACAAGCTCTTGAACTTCGTGGTAACACTAGAATTGACTCTCGTGGCGCTGATACGAACACACCTTTGGAATATTTATCAAAGTACGCCATTGATATGACTGAGCAGGCTCGTCAAGGTAAACTTGACCCTGTCATCGGCCGTGAAGAAGAAATAAGAAGCACTATTAGAGTTTTAGCAAGAAGAATTAAGTCCAACCCATGTTTAATTGGTGAGCCAGGTATCGGTAAGACCGCTATTATTGAAGGTGTTGCTCAAAGAATCATTGACGATGACGTTCCCACTATCTTACAAGGCGCTAAATTGTTCAGTCTAGATTTGGCCGCATTAACCGCAGGTGCTAAATACAAAGGTGATTTCGAAGAAAGATTCAAAGGTGTTTTGAAGGAAATCGAAGAATCAAAGACTCTAATTGTGTTATTCATTGATGAAATTCACATGTTAATGGGTAATGGTAAGGACGACGCTGCTAACATCTTGAAGCCAGCTTTGTCCAGAGGCCAATTGAAGGTCATCGGTGCCACCACCAATAACGAATATAGATCTATTGTGGAAAAGGATGGTGCCTTTGAAAGAAGATTCCAGAAAATTGAAGTCGCTGAACCAAGTGTGAGACAAACAGTGGCCATATTGAGAGGTCTGCAACCAAAGTATGAAATACATCATGGTGTAAGGATTCTGGATAGCGCCTTAGTCACTGCTGCTCAATTAGCCAAGCGTTACTTGCCATATAGAAGATTGCCAGATTCTGCTTTGGATTTAGTTGATATTTCTTGTGCTGGTGTCGCCGTCGCAAGAGATTCTAAGCCAGAAGAATTGGATTCCAAGGAACGTCAATTGCAATTGATTCAAGTAGAGATAAAAGCTCTAGAGAGAGATGAAGATGCCGACTCCACCACTAAAGATAGATTAAAGTTAGCTAGGCAGAAGGAAGCTTCATTGCAAGAAGAATTGGAACCTCTAAGACAACGTTACAATGAAGAAAAGCATGGCCATGAAGAATTGACACAAGCTAAAAAGAAATTGGATGAACTGGAAAACAAGGCCCTTGATGCTGAACGTAGATATGATACTGCTACCGCCGCTGATTTAAGGTACTTCGCCATCCCAGATATCAAAAAGCAAATCGAAAAGCTTGAAGATCAGGTTGCTGAGGAAGAGAGACGTGCTGGTGCCAACTCCATGATCCAAAATGTGGTCGATTCAGACACCATTTCTGAAACAGCTGCAAGATTGACTGGTATCCCTGTTAAGAAGTTGTCAGAATCTGAAAATGAAAAATTGATTCATATGGAACGTGACTTATCATCTGAAGTCGTGGGCCAAATGGATGCCATTAAAGCTGTTTCCAATGCCGTTAGATTGTCTAGATCAGGTTTAGCTAATCCAAGGCAACCAGCATCCTTCTTATTTTTAGGTTTGTCCGGTTCCGGTAAAACTGAATTGGCTAAAAAAGTTGCTGGATTTTTGTTTAATGATGAGGACATGATGATCAGGGTCGATTGTTCTGAATTAAGCGAGAAGTATGCGGTCTCTAAGTTGTTGGGTACCACGGCAGGTTATGTCGGGTACGATGAAGGTGGCTTTTTAACTAACCAACTGCAATACAAACCATACTCCGTTTTGTTATTCGATGAAGTAGAAAAGGCACATCCTGATGTTTTGACTGTCATGCTACAAATGTTGGATGACGGTAGAATTACTTCTGGTCAAGGTAAGACGATCGACTGTTCCAATTGTATTGTCATCATGACTTCCAATCTAGGTGCTGAATTTATCAATTCTCAACAAGGATCAAAGATCCAAGAATCTACCAAGAATTTGGTCATGGGTGCTGTTAGGCAACATTTCAGACCAGAATTTTTGAACAGAATTTCTAGTATAGTCATTTTCAACAAGCTATCTAGAAAAGCTATTCATAAGATCGTGGATATTCGTTTGAAGGAAATTGAAGAGAGATTCGAGCAAAATGATAAACATTACAAGTTGAATTTAACTCAAGAGGCCAAGGACTTCTTGGCCAAATATGGTTATTCCGATGATATGGGTGCACGTCCACTGAACAGGTTAATTCAAAACGAAATTTTGAACAAACTGGCACTAAGGATCTTAAAGAATGAAATCAAGGATAAGGAAACTGTCAATGTCGTCTTGAAGAAGGGTAAATCTCGTGATGAAAATGTTCCTGAGGAAGCTGAAGAATGTCTGGAAGTTCTACCAAATCACGAAGCTACTATAGGGGCTGACACGTTAGGTGATGACGATAATGAGGACAGTATGGAAATTGATGATGACCTAGATTAA |
| Hul5 | ATGTTAAACTTCACCGGTCAAACAAGGAGAAGAAATGTCAATTTAGGGAATAGGACTCGAAATTCAAAGAAGGATTTACTGGAAAAGGCCAAAAGGGAACGTGAAAGGAGAGCACAAGATAAGCTCAAAGAAGACGCCAGTAAAACCATTCAAAAAAGCATCAGAAGACATTTTTCAAATGTGAGACTCTTCAAAAACACATTTACTAGTTCGCAACTTGTTCATATGATACCAGCTTACGGGGGCAAATTAATCTATTACATTTCTCAATATGATCTGCAGCAACTGCTAAAATTATCTCATAATTTTTTGAGTTCTTATCCTAATTCTTTAGGCAACAGACAGCTATTGAGCTTGTTGAAGCTATATCAAGATGATGCACTGGTGGCTGAAACTCTAAGCGATCTTAACATGGACTGCCCTACAGTTGACGAATTTTTAGATAGTCTATCCGTTTATCTCTGTCGGGCTTCCTCCTTGAGTTATTCCTCAGCTTCTAAGCTAGCTGATGTCATAGAAGCATGGGAAGTAATGCATAGCAGTGCCTCCATTAGTATTTTTTCGATATCGATAGGATCTTACGAAAAACGGCCATTTGCACTACAGTTTTATTGCATACTTGCCGAAAGAAACCTTTTGCCCCAACTTATCAACACAAATCCGATATTATGGGATAACATGGCAAAGACATATTCACACTGTAGTAAAGGTGGCCAAAAAAATATCGCCAAGCTACTCATACCAAACTTCAACAATCATATTGCTCCATCAGTCTTGCGTAGTGATAACGACTATGTCTTGAAATTTTATGAAAAGGCATTTATAGACGAAGTTATTGCTACTACTGCAAATTACGTTTCTGATGAAGATCACGTGAAGAATTTGATGTGCTACATTGCAAGCTCTCCCAATCAAAGCTGTAAGAATTCTGTCCTTATTACCTTACTGTCTAACAAAGACTTCGTGAGAAGGCTCTCATGGGAATTCTTTCACACTAAATTCAACGCCAGCAAGACTGAGGCTCACCCCCTGTTTTCAGTTTTGGCACAGCTTATCGACATGCACCTTTTAATATCGACAGATCGGGAGTTATTAGACTACAACTCTGTGATACCTATCGAAGAACTAAAGAAATTTACATCCACGTTAAAAGATTTTACTTTCCGACAATATTGGGAACTACCTAAAAGTGAAAGGAACCCTATGTTAAAGGAAGCAGTACCACTTTTGAGCAAAGTCTACGAAAGGGACTCAAGATTGCACTTTCTATCCACGGAGAATAATCCAACCTATTGGGAAAACTCTGAAAAGCAATTTTTAAATTTGAGATTTTACGAAGAATTACAGGAGTACGAAGATCTGTATAGAGAACACTTAGAAGAGGAAAGTGATGAGGATATGGAAAAGGAAATAGATCTTGATAAAGAAAGGCCTCCTTTGAAGTCTTTATTATTGAACAAAATGAAAAAGAGATTAAAGTCATCATTACGTTTCCGAAAGTTGGAAATACTTTTGGAATTACCATTTTTTATTCCTTTTGAAGAAAGGGTGGACTTATTTTATATGTTCATCGCACTGGACAAGAAGCGATTATCTTTAGATGACGATCACAACTTGATCAATATGTTTACCCCCTGGGCCTCCACCGGTATGAGGAAGCAATCCGCTATCATCTCTAGGGATAATGTCTTAGAAGATGCTTTCAACGCATTTAACTCTATAGGAGAAAGGTTCAAAGCTTCATTAGATGTTACTTTTATTAATGAATTTGGTGAAGAAGCTGGTATTGATGGGGGCGGCATTACCAAGGAATTTTTAACTACTGTGTCTGATGAAGGATTTAAAGATCCAAAGCACGAGTTATTTCGGACGAATGATCGCTACGAATTATATCCTTCTGTTGTTTATGACGCTACGAAACTTAAGTATATATGGTTTCTGGGAAAGGTTGTAGGCAAATGTCTATATGAGCATGTTTTGATAGATGTATCTTTTGCTGATTTCTTTTTGAAAAAATTATTGAATTACTCGAACGGGTTTTTATCTTCTTTTTCTGATCTGGGAAGCTATGATTCGGTGTTGTATAATAATTTGATCAAATTATTGAACATGACCACTGACGAGATCAAGTCTTTAGATTTAACCTTTGAAATAGATGAGCCTGAAAGTTCCGCAAAAGTTGTTGACTTAATTCCGAATGGTTCAAAAACGTACGTGACGAAGGATAATGTGTTGCTCTACGTTACTAAAGTAACGGATTACAAATTAAACAAAAGATGCTTCAAACCAGTTTCGGCATACCATGGAGGGCTCAGTGTTATCATTGCCCCACATTGGATGGAGATGTTTAACTCTATAGAACTACAAATGTTAATATCAGGTGAGAGAGATAATATCGATTTAGACGATCTGAAATCTAACACAGAATACGGGGGTTATAAAGAAGAAGATCAGACAATTGTTGATTTTTGGGAGGTTTTGAATGAGTTTAAGTTTGAAGAGAAATTGAATTTTTTGAAATTTGTCACGTCCGTTCCGCAAGCTCCTCTGCAAGGCTTCAAGGCATTAGATCCAAAATTTGGTATCAGAAATGCAGGGACAGAGAAATACAGGCTGCCTACGGCATCTACCTGTGTTAATTTATTAAAATTGCCAGATTATAGAAACAAAACAATTTTGAGAGAGAAATTATTATATGCAATAAACTCAGGCGCCAGGTTTGACTTATCATAA |
| Msn2 (SC) | ATGACGGTCGACCATGATTTCAATAGCGAAGATATTTTATTCCCCATAGAAAGCATGAGTAGTATACAATACGTGGAGAATAATAACCCAAATAATATTAACAACGATGTTATCCCGTATTCTCTAGATATCAAAAACACTGTCTTAGATAGTGCGGATCTCAATGACATTCAAAATCAAGAAACTTCACTGAATTTGGGGCTTCCTCCACTATCTTTCGACTCTCCACTGCCCGTAACGGAAACGATACCATCCACTACCGATAACAGCTTGCATTTGAAAGCTGATAGCAACAAAAATCGCGATGCAAGAACTATTGAAAATGATAGTGAAATTAAGAGTACTAATAATGCTAATGGCTCTGGGGCAAATCAATACACAACTCTTACTTCACCTTATCCTATGAACGACATTTTGTACAACATGAACAATCCGTTACAATCACCGTCACCTTCATCGGTACCTCAAAATCCGACTATAAATCCTCCCATAAATACAGCAAGTAACGAAACTAATTTATCGCCTCAAACTTCAAATGGTAATGAAACTCTTATATCTCCTCGAGCCCAACAACATACGTCCATTAAAGATAATCGTCTGTCCTTACCTAATGGTGCTAATTCGAATCTTTTCATTGACACTAACCCAAACAATTTGAACGAAAAACTAAGAAATCAATTGAACTCAGATACAAATTCATATTCTAACTCCATTTCTAATTCAAACTCCAATTCTACGGGTAATTTAAATTCCAGTTATTTTAATTCACTGAACATAGACTCCATGCTAGATGATTACGTTTCTAGTGATCTCTTATTGAATGATGATGATGATGACACTAATTTATCACGCCGAAGATTTAGCGACGTTATAACAAACCAATTTCCGTCAATGACAAATTCGAGGAATTCTATTTCTCACTCTTTGGACCTTTGGAACCATCCGAAAATTAATCCAAGCAATAGAAATACAAATCTCAATATCACTACTAATTCTACCTCAAGTTCCAATGCAAGTCCGAATACCACTACTATGAACGCAAATGCAGACTCAAATATTGCTGGCAACCCGAAAAACAATGACGCTACCATAGACAATGAGTTGACACAGATTCTTAACGAATATAATATGAACTTCAACGATAATTTGGGCACATCCACTTCTGGCAAGAACAAATCTGCTTGCCCAAGTTCTTTTGATGCCAATGCTATGACAAAGATAAATCCAAGTCAGCAATTACAGCAACAGCTAAACCGAGTTCAACACAAGCAGCTCACCTCGTCACATAATAACAGTAGCACTAACATGAAATCCTTCAACAGCGATCTTTATTCAAGAAGGCAAAGAGCTTCTTTACCCATAATCGATGATTCACTAAGCTACGACCTGGTTAATAAGCAGGATGAAGACCCCAAGAACGATATGCTGCCGAATTCAAATTTGAGTTCATCTCAACAATTTATCAAACCGTCTATGATTCTTTCAGACAATGCGTCCGTTATTGCGAAAGTGGCGACTACAGGCTTGAGTAATGATATGCCATTTTTGACAGAGGAAGGTGAACAAAATGCTAATTCTACTCCAAATTTCGATCTTTCCATCACTCAAATGAATATGGCTCCATTATCGCCTGCATCATCATCCTCCACGTCTCTTGCAACAAATCATTTCTATCACCATTTCCCACAGCAGGGTCACCATACCATGAACTCTAAAATCGGTTCTTCCCTTCGGAGGCGGAAGTCTGCTGTGCCTTTGATGGGTACGGTGCCGCTTACAAATCAACAAAATAATATAAGCAGTAGTAGTGTCAACTCAACTGGCAATGGTGCTGGGGTTACGAAGGAAAGAAGGCCAAGTTACAGGAGAAAATCAATGACACCGTCCAGAAGATCAAGTGTCGTAATAGAATCAACAAAGGAACTCGAGGAGAAACCGTTCCACTGTCACATTTGTCCCAAGAGCTTTAAGCGCAGCGAACATTTGAAAAGGCATGTGAGATCTGTTCACTCTAACGAACGACCATTTGCTTGTCACATATGCGATAAGAAATTTAGTAGAAGCGATAATTTGTCGCAACACATCAAGACTCATAAAAAACATGGAGACATTTAA |
| Msn4 | ATGCTAGTCTTCGGACCTAATAGTAGTTTCGTTCGTCACGCAAACAAGAAACAAGAAGATTCGTCTATAATGAACGAGCCAAACGGATTGATGGACCCGGTATTGAGCACAACCAACGTTTCTGCTACTTCTTCTAATGACAATTCTGCGAACAATAGCATATCTTCGCCGGAATATACCTTTGGTCAATTCTCAATGGATTCTCCGCATAGAACGGACGCCACTAATACTCCAATTTTAACAGCGACAACTAATACGACTGCTAATAATAGTTTAATGAATTTAAAGGATACCGCCAGTTTAGCTACCAACTGGAAGTGGAAAAATTCCAATAACGCACAGTTCGTGAATGACGGTGAGAAACAAAGCAGTAATGCTAATGGTAAGAAAAATGGTGGTGATAAGATATATAGTTCAGTAGCCACCCCTCAAGCTTTAAATGACGAATTGAAAAACTTGGAGCAACTAGAAAAGGTATTTTCTCCAATGAATCCTATCAATGACAGTCATTTTAATGAAAATATAGAATTATCGCCACACCAACATGCAACTTCTCCCAAGACAAACCTTCTTGAGGCAGAACCTTCAATATATTCCAATTTGTTTCTAGATGCTAGGTTACCAAACAACGCCAACAGTACAACAGGATTGAACGACAATGATTATAATCTAGACGATACCAATAATGATAATACTAATAGCATGCAATCAATCTTAGAGGATTTTGTATCTTCAGAAGAAGCATTGAAGTTCATGCCGGACGCTGGTCGCGACGCAAGAAGATACAGCGAGGTGGTTACCTCTTCCTTTCCTTCTATGACGGATTCTAGAAATTCGATCTCTCATTCGATAGAGTTTTGGAATCTCAATCACAAAAATAGTAGCAACAGTAAACCCACTCAACAAATTATCCCTGAAGGTACTGCCACTACTGAGAGGCGTGGATCAACCATTTCACCTACTACCACTATAAACAACTCTAATCCAAACTTCAAATTATTAGATCATGACGTTTCTCAAGCTCTGAGCGGTTATAGTATGGATTTTTCTAAGGACTCTGGTATAACAAAGCCAAAAAGCATTTCCTCTTCTTTAAATCGCATCTCCCATAGCAGTAGCACCACAAGGCAACAGCGTGCCTCTTTGCCCTTAATTCATGATATTGAATCTTTTGCAAATGATTCGGTGATGGCAAATCCTCTGTCTGATTCCGCATCATTTCTTTCAGAAGAAAATGAAGACGATGCTTTTGGTGCGCTAAATTACAATAGCTTAGATGCAACCACAATGTCGGCATTCGACAATAACGTAGACCCCTTCAACATTCTCAAGTCATCTCCGGCTCAGGATCAACAGTTTATCAAACCCTCTATGATGTTGTCGGATAATGCCTCTGCTGCCGCTAAATTGGCGACTTCTGGTGTTGATAATATCACACCTACACCAGCTTTCCAAAGAAGAAGCTATGATATCTCGATGAACTCTTCGTTCAAAATACTTCCTACTAGTCAAGCTCACCATGCAGCTCAACATCATCAACAACAACCTACTAAACAGGCAACGGTAAGCCCAAACACAAGAAGAAGAAAGTCGTCAAGTGTTACTTTAAGTCCAACTATTTCTCATAACAACAACAATGGTAAGGTTCCTGTCCAACCTCGGAAAAGGAAATCTATTACTACCATTGACCCCAACAACTACGATAAAAATAAACCTTTCAAGTGTAAAGACTGTGAGAAGGCATTCAGACGCAGTGAGCACTTGAAAAGGCATATAAGATCCGTTCATTCAACGGAACGCCCTTTTGCTTGTATGTTCTGTGAGAAAAAATTCAGTAGAAGTGACAATTTATCACAACATCTAAAAACTCACAAAAAGCACGGTGATTTTTGA |
| Pma1 | ATGACTGATACATCATCCTCTTCATCATCCTCTTCAGCATCTTCTGTTTCAGCTCATCAGCCAACTCAAGAAAAGCCTGCTAAGACTTACGATGACGCTGCATCTGAATCTTCTGACGATGACGATATCGATGCTTTAATCGAAGAACTACAATCTAATCACGGTGTCGACGACGAAGACAGTGATAACGATGGTCCAGTTGCCGCCGGTGAAGCTAGACCAGTTCCAGAAGAATATTTACAAACTGACCCATCTTACGGTTTAACTTCCGATGAAGTTTTGAAAAGAAGAAAGAAGTACGGTTTGAATCAAATGGCTGACGAAAAAGAATCTTTAGTCGTTAAGTTCGTTATGTTTTTCGTCGGTCCAATTCAATTCGTTATGGAAGCCGCTGCTATTTTGGCTGCCGGTTTGTCCGATTGGGTCGATTTCGGTGTTATCTGTGGTTTGTTAATGTTAAACGCTGGTGTTGGTTTCGTTCAAGAATTCCAAGCTGGTTCTATTGTCGATGAATTGAAAAAGACTTTGGCTAACACTGCTGTTGTTATCAGAGACGGTCAATTGGTTGAAATTCCAGCCAACGAAGTCGTCCCAGGTGATATTTTGCAATTGGAAGATGGTACTGTTATCCCAACTGATGGTCGTATTGTCACTGAAGACTGTTTCTTGCAAATCGATCAATCTGCTATTACTGGTGAATCTTTGGCTGTCGACAAACATTACGGTGACCAAACTTTCTCTTCTTCCACTGTTAAGAGAGGTGAAGGTTTCATGGTTGTTACCGCTACCGGTGACAACACTTTCGTTGGTAGAGCTGCTGCTTTGGTTAACAAAGCCGCTGGTGGTCAAGGTCATTTCACTGAAGTTTTGAACGGTATTGGTATTATCTTATTGGTTTTGGTCATTGCCACTTTGTTGTTGGTCTGGACTGCTTGTTTCTACAGAACCAACGGTATTGTTAGAATCTTGAGATACACTCTAGGTATTACTATTATTGGTGTCCCAGTCGGTTTGCCAGCTGTCGTTACCACCACTATGGCCGTCGGTGCTGCTTACTTGGCTAAGAAACAAGCCATTGTTCAAAAGTTGTCTGCTATTGAATCCTTGGCTGGTGTCGAAATCTTGTGTTCCGACAAAACCGGTACTTTGACCAAGAACAAGTTGTCCTTGCACGAACCATACACTGTTGAAGGTGTTTCTCCAGACGACTTGATGTTGACTGCTTGTTTGGCTGCTTCCAGAAAGAAGAAGGGTTTGGATGCTATTGATAAGGCTTTCTTGAAGTCTTTGAAGCAATATCCAAAGGCTAAGGACGCTTTGACCAAGTACAAGGTTTTGGAATTCCATCCATTTGACCCTGTCTCCAAGAAGGTTACTGCCGTTGTCGAATCTCCAGAAGGTGAAAGAATTGTTTGTGTTAAGGGTGCTCCATTATTCGTCTTGAAGACTGTCGAAGAAGATCACCCAATCCCAGAAGATGTCCATGAAAACTACGAAAACAAGGTTGCTGAATTGGCTTCTAGAGGGTTCCGTGCTTTAGGTGTTGCTAGAAAGAGAGGTGAAGGTCACTGGGAAATCTTGGGTGTTATGCCATGTATGGATCCACCAAGAGACGATACTGCTCAAACTGTTAGCGAAGCTAGACACTTGGGTCTAAGAGTTAAGATGTTAACTGGTGATGCTGTTGGTATTGCTAAGGAAACTTGTAGACAATTGGGTTTGGGTACCAACATTTACAACGCTGAAAGATTAGGTCTAGGTGGCGGTGGTGACATGCCAGGTTCTGAATTAGCTGATTTTGTTGAAAATGCCGATGGTTTCGCTGAAGTTTTCCCACAACATAAATACAGAGTTGTTGAAATCTTGCAAAACAGAGGTTACTTGGTTGCTATGACTGGTGATGGTGTTAACGATGCTCCATCTTTGAAGAAGGCTGATACTGGTATTGCTGTCGAAGGTGCTACTGATGCTGCCAGATCTGCTGCTGATATTGTTTTCTTGGCTCCTGGTCTATCTGCTATTATTGATGCTTTGAAGACCTCCAGACAAATTTTTCACAGAATGTACTCTTACGTTGTTTACCGTATTGCTTTGTCTCTACATTTGGAAATCTTCTTGGGTCTATGGATTGCTATTTTGGATAACTCTTTGGACATTGATTTGATTGTTTTCATCGCTATTTTCGCTGATGTTGCTACTTTGGCTATTGCTTACGATAATGCTCCTTACTCTCCAAAGCCCGTTAAATGGAACCTACCAAGATTATGGGGTATGTCTATTATTTTGGGCATAGTTTTAGCTATAGGTTCTTGGATTACCTTGACTACTATGTTCTTACCAAAGGGTGGTATTATCCAAAACTTCGGTGCTATGAACGGTATTATGTTCTTGCAAATTTCCTTGACTGAAAACTGGTTGATTTTCATTACCAGAGCTGCTGGTCCATTCTGGTCTTCTATCCCATCCTGGCAATTGGCTGGTGCCGTCTTCGCTGTCGACATCATCGCTACCATGTTTACCTTATTCGGTTGGTGGTCTGAAAACTGGACTGATATTGTTACTGTCGTCCGTGTCTGGATCTGGTCTATCGGTATCTTCTGTGTTTTGGGTGGTTTCTACTACGAAATGTCCACTTCTGAAGCCTTTGACAGATTGATGAACGGTAAGCCAATGAAGGAAAAGAAGTCTACCAGAAGTGTCGAAGACTTCATGGCTGCTATGCAAAGAGTCTCTACTCAACACGAAAAGGAAACCTAA |
| Prx1 | ATGTTTAGTAGAATTTGTAGCGCTCAATTAAAGAGGACGGCATGGACCCTTCCCAAGCAGGCTCACTTGCAATCACAGACGATTAAAACATTTGCCACAGCACCTATTCTGTGCAAACAATTCAAACAAAGTGATCAACCAAGACTAAGAATAAACTCTGATGCTCCTAACTTTGATGCTGACACAACGGTTGGTAAAATCAATTTTTACGACTACTTGGGCGACTCTTGGGGGGTCTTGTTTTCTCACCCAGCAGATTTCACCCCTGTCTGCACCACCGAAGTCAGCGCATTCGCCAAATTGAAGCCGGAATTCGACAAGAGAAATGTTAAATTGATCGGGCTTTCAGTGGAAGATGTTGAGTCCCACGAAAAATGGATTCAAGACATCAAGGAAATAGCAAAGGTTAAAAATGTTGGTTTCCCAATAATTGGTGACACTTTTAGAAACGTGGCATTCCTATATGATATGGTAGATGCCGAAGGATTCAAAAATATCAATGATGGGTCACTGAAGACCGTGAGGTCTGTTTTCGTCATCGATCCCAAGAAGAAGATTAGACTGATTTTTACCTACCCTTCCACCGTCGGAAGAAACACTTCTGAAGTGTTAAGGGTAATCGACGCCTTGCAATTGACTGACAAGGAGGGCGTAGTAACTCCAATTAATTGGCAGCCAGCTGACGATGTCATTATTCCTCCCTCTGTCTCCAATGATGAGGCGAAGGCTAAATTTGGTCAATTCAATGAAATTAAACCCTATTTAAGATTCACCAAGTCGAAATAA |
| San1 | ATGAGTGAAAGTGGTCAAGAACAAGACAGAGGCACAAATACATCACCAAATAATGCTGAAAATAATAATAATTCAAATGCAGCTTCCGGTCCACTCAATGGTGGTGCTGAGCAAACAAGAAACATAACCGTTTCCATTCAGTATTCCTATTTCACTCCGGAGAGATTAGCACATTTGAGCAATATATCTAATAATGATAATAACGAAAACAACAGCGCAGCATCCGGTAGCACGATTGCCAACGGTACTGGGCCCAGCTTTGGCATCGGAAATGGGGGCCATCAACCCGACGGTGCTCTTGTTTTATCTTTTCGTGATGTTCCTGCGTCTACTCCACAAGACCGTTTGAACAGCTTCATATCTGTTGCTGCTCAGCTTGCAATGGAGAGATTCAACAGACTATTAAATAGACCAAAAGGAATATCAAAGGATGAGTTTGATAAGCTCCCCGTTCTGCAAGTTTCTGATCTACCCAAGGCCGAAGGGCCCTTATGTAGTATATGTTATGACGAATATGAAGATGAAGTTGATTCAACTAAAGCAAAAAGAAAAAGGGATTCTGAAAATGAGGAGGAATCTGAAGGAACAAAAAAAAGGAAGGATAATGAAGGTGCGCCCCTACGCACAACCGCCGATAATGACAGTAACCCATCGATTACAAATGCTACGGTTGTTGAACCGCCTTCTATTCCTCTCACTGAACAACAACGCACTCTCAATGATGAAGAAACAAATCCAAGCTACAAACACTCACCAATCAAGTTACCTTGTGGCCACATTTTTGGGAGGGAATGTATCTACAAATGGTCAAGATTAGAAAATTCTTGTCCCCTTTGTAGACAAAAGATCAGCGAATCTGTAGGTGTTCAACGTGCAGCCCAACAAGATACGGATGAAGTAGCAGCTAACGAAGCTGCTTTTGAACGTATTAGACGAGTTTTATACGACCCAACTGCAGTGAATAGCACTAACGAAAATAGTTCCGCCCCATCCGAAAACACGTCCAATACAACGGTTCACACTATCGGAAATGCAAGTTCTGGCGAACAGATGTTATCAAGAACAGGCTTTTTTTTAGTGCCTCAAAACGGCCAACCTTTACACAATCCAGTCCGTTTACCGCCTAATGATAGCGATAGAAACGGTGTCAACGGGCCGAGCTCAACTACTCAAAATCCACCCTCTAATTCTGGTGGTTCGAATAACAATCAAAGTCCGCGCTGGGTTCCCATCCCTTTGACTTTGTTCCAATTCCACAGTCCAAATCCGAATCCTTCCGCTTCTGATTCTTCGGCAAGCCCATCAGCAGCGAACGGTCCAAACTCAAATAACACTAGTAGTGACGCTACAGACCCTCACCACAACAGACTAAGAGCCGTTTTGGATCACATATTCAACGTTGCTCAGAGGGGAACTTCTGATACCTCTGCAACAACAGCACCCGGAGCACAAACTGTTCACAACCAAGGACGTAATGACTCATCGTCCTCTGATACAACGCAAGGAAGTTCCTTTTTGGAAAATATTTCACGATTAACAGGCCATTTTACGAATGGCTCAAGAGACAACAATAACGACAATAACCATAGCAATGATCAACAACGAGGTGGAAGTACTGGTGAGAACAACAGAAATAACTTGTTTTCCTCCGGTGTTGCCAGTTATAGAAATCAAAATGGTGATGTTACTACCGTCGAACTACGCAACAACAATTCTGCTGCCTTTCCTCCTACAGACGAAAATCCCTCTCAAGGCCAAGGTTCAAGCAGTTCGGACACCACCATTCATAACGACGTCCCTAATGATAACAATGAGCAACGATCATCACAATAA |
| Skn7 | ATGAGCTTTTCCACCATAAATAGCAACGTCAATAAAACCACCGGCGATAGCAATAATAACACCACCGAGAACAGTTCGACTGCAGACCTTTTAGGAATGGACTTGTTGCAGAGCGGGCCTCGACTGATGAACACGATGCAGCCAAACAACTCTTCTGACATGCTGCACATTAACAACAAGACTAATAACGTTCAACAACCAGCTGGAAACACAAATATCAGCAGTGCTAATGCGGGAGCAAAGGCTCCAGCAAATGAGTTCGTAAGAAAACTGTTCAGGATACTGGAAAACAATGAATATCCTGACATTGTAACTTGGACTGAGAACGGCAAAAGTTTCGTCGTTTTGGACACAGGAAAGTTCACTACGCATATATTGCCTAATCACTTCAAACATTCAAATTTTGCATCTTTTGTAAGGCAACTAAACAAGTATGACTTTCACAAGGTTAAGAGAAGTCCCGAGGAAAGACAGAGATGTAAATATGGCGAACAAAGTTGGGAGTTTCAGCATCCAGAATTTAGAGTCCATTACGGAAAAGGTCTCGATAACATCAAAAGGAAAATTCCGGCGCAAAGGAAAGTGCTTTTGGATGAATCTCAAAAGGCTCTTTTGCATTTCAATAGTGAAGGCACTAACCCCAACAATCCTTCTGGGTCTCTTTTGAATGAATCCACCACAGAGCTGTTGTTAAGCAATACCGTAAGTAAAGATGCATTTGGAAATCTAAGAAGGCGAGTAGACAAACTACAAAAGGAGTTGGATATGTCCAAAATGGAGAGTTATGCTACTAAAGTAGAACTACAAAAGTTGAACTCGAAATACAATACGGTTATCGAAAGTTTGATAACATTCAAGACCATAAATGAAAATTTACTCAACAACTTCAACACTCTGTGTTCCACTTTGGCAAATAATGGTATTGAAGTGCCAATATTTGGCGACAATGGAAACCGTAACCCAACTGGTAATACCAACCCAGCAACAACAACAGCTATCCAAAGCAACAACAACACCAACAATGCTTCTCCGGCAACATCTACAGTTTCCTTACAACTACCTAATTTACCCGATCAGAATAGCCTAACACCAAATGCTCAAAATAACACAGTCACGCTACGAAAAGGTTTCCATGTACTGTTGGTGGAAGATGACGCAGTGTCTATACAGTTGTGTTCAAAATTTTTACGGAAATATGGCTGTACTGTCCAAGTTGTCAGCGACGGTCTTTCAGCTATCTCAACACTAGAGAAGTATAGGTATGATTTGGTTTTAATGGACATTGTTATGCCAAACCTAGATGGTGCCACAGCGACATCCATTGTCAGAAGTTTTGATAATGAGACTCCCATCATTGCCATGACAGGTAACATTATGAATCAAGACTTGATCACATACTTACAACATGGAATGAATGATATCTTGGCCAAACCATTCACGAGGGATGATTTACACTCAATTTTAATACGTTATCTAAAGGACCGTATTCCTTTATGCGAACAGCAATTACCACCTCGCAACTCTTCGCCACAAACTCATTCCAACACCAATACTGCTAATTCGAATCCTAATACGATTAATGAACAGTCGTTAGCCATGTTACCACAAGATAATCCGTCAACTACTACCCCTGTTACCCCAGGTGCCTCTATATCTTCTGCACAGCATGTTCAACAAGGTCAACAAGAACAGCAGCATCAAATTTTCCATGCTCAGCAGCAGCAGCAGCATCACAACGCCATTGCTAATGCTAGGTCAGACGTAGCCATACCGAATTTGGAACATGAAATCAACACTGTACCACATTCCTCAATGGGTTCCACTCCGCAATTACCACAATCTACACTTCAAGAAAACCAGCTATCATAA |
| Sod1 | ATGGTTCAAGCAGTCGCAGTGTTAAAGGGTGATGCCGGTGTCTCTGGTGTTGTCAAGTTCGAACAGGCTTCCGAATCCGAGCCAACCACTGTCTCTTACGAGATCGCTGGTAACAGTCCTAACGCAGAACGTGGGTTCCACATTCATGAGTTTGGAGATGCCACCAATGGTTGTGTCTCTGCTGGTCCTCACTTCAATCCTTTCAAGAAGACACATGGTGCTCCAACTGACGAAGTCAGACATGTCGGTGACATGGGTAACGTAAAGACGGACGAAAATGGTGTGGCCAAGGGCTCCTTCAAGGACTCTTTGATCAAGCTTATCGGTCCTACCTCCGTTGTAGGCAGAAGCGTCGTTATCCACGCCGGCCAAGATGACTTAGGTAAGGGTGACACTGAAGAATCTTTGAAGACTGGTAATGCCGGTCCAAGACCAGCCTGTGGTGTCATTGGTCTAACCAACTAA |
| Tsa1 | ATGGTCGCTCAAGTTCAAAAGCAAGCTCCAACTTTTAAGAAAACTGCCGTCGTCGACGGTGTCTTTGACGAAGTCTCCTTGGACAAATACAAGGGTAAGTACGTTGTCCTAGCCTTTATTCCATTGGCCTTCACTTTCGTCTGTCCAACCGAAATCATTGCTTTCTCAGAAGCTGCTAAGAAATTCGAAGAACAAGGCGCTCAAGTTCTTTTCGCCTCCACTGACTCCGAATACTCCCTTTTGGCATGGACCAATATCCCAAGAAAGGAAGGTGGTTTGGGCCCAATCAACATTCCATTGTTGGCTGACACCAACCACTCTTTGTCCAGAGACTATGGTGTCTTGATCGAAGAAGAAGGTGTCGCCTTGAGAGGTTTGTTCATCATCGACCCAAAGGGTGTCATTAGACACATCACCATTAACGATTTGCCAGTCGGTAGAAACGTTGACGAAGCCTTGAGATTGGTTGAAGCCTTCCAATGGACCGACAAGAACGGTACTGTCTTGCCATGTAACTGGACTCCAGGTGCTGCTACCATCAAGCCAACCGTTGAAGACTCCAAGGAATACTTCGAAGCTGCCAACAAATAA |
| Vma2 | ATGGTTTTGTCTGATAAGGAGTTGTTTGCCATAAATAAGAAAGCCGTCGAACAAGGTTTCAATGTGAAGCCTAGATTGAACTATAATACGGTCAGTGGTGTGAACGGTCCATTAGTCATTTTGGAAAAGGTCAAGTTCCCACGTTACAACGAAATTGTTAATTTGACATTGCCAGATGGAACCGTGAGACAAGGTCAAGTTTTGGAAATTAGAGGAGATAGAGCCATTGTGCAAGTGTTTGAAGGTACATCTGGTATTGATGTCAAGAAGACTACCGTGGAATTCACTGGTGAGAGTTTGAGAATTCCTGTGTCTGAAGACATGTTGGGTAGAATTTTTGACGGTTCTGGTAGACCCATTGACAACGGTCCTAAAGTTTTCGCAGAGGATTACTTGGACATTAACGGTTCTCCTATCAACCCATATGCTCGTATTTATCCAGAAGAAATGATTTCTACTGGTGTTTCTGCTATTGACACAATGAACTCCATTGCCAGAGGTCAAAAGATCCCAATTTTCTCCGCATCAGGTTTACCACACAACGAAATTGCAGCACAAATTTGTAGACAGGCTGGTTTGGTGAGACCTACCAAGGATGTTCATGATGGTCATGAAGAAAATTTCTCCATCGTTTTTGCTGCCATGGGTGTCAACTTGGAAACCGCTAGATTTTTCAAACAGGATTTCGAAGAAAATGGGTCTTTGGAAAGAACTTCATTATTTTTGAACTTGGCTAATGACCCTACCATTGAAAGAATTATCACTCCAAGATTGGCCTTGACCACCGCTGAATACCTTGCTTACCAAACGGAACGTCATGTGTTGACCATCTTGACCGATATGTCATCGTATGCTGATGCTCTTAGAGAAGTTTCCGCTGCTAGAGAAGAAGTTCCAGGTAGAAGAGGTTATCCTGGTTACATGTATACAGATTTGTCCACAATTTATGAAAGAGCAGGTAGAGTAGAGGGTCGTAACGGGTCCATCACTCAAATACCTATCTTGACAATGCCTAACGATGATATTACGCATCCAATTCCGGATTTGACCGGTTATATTACCGAGGGTCAAATCTTCGTTGACCGTCAATTACATAACAAGGGTATCTACCCACCAATCAACGTCTTGCCTTCGTTGAGTAGATTGATGAAATCTGCCATCGGTGAAGGTATGACCAGAAAGGACCACGGTGACGTTTCTAACCAATTGTATGCCAAGTACGCCATCGGTAAGGACGCTGCTGCTATGAAGGCCGTTGTCGGTGAAGAGGCGTTATCCATCGAAGATAAGTTATCTTTGGAATTTTTGGAAAAATTCGAAAAGACCTTTATCACACAAGGCGCCTACGAGGACAGAACCGTTTTCGAAAGTTTGGACCAGGCATGGAGTTTGCTAAGAATCTACCCTAAGGAGATGTTGAATAGAATCTCCCCAAAGATTCTTGATGAATTTTACGATAGAGCCAGAGACGATGCCGACGAAGATGAAGAAGATCCCGACACAAGAAGCTCCGGTAAGAAGAAGGACGCCAGCCAAGAAGAATCTCTAATCTAA |
| Vma3 | ATGACTGAATTGTGTCCTGTCTACGCCCCTTTCTTTGGTGCCATTGGTTGTGCCTCTGCAATTATCTTCACCTCATTAGGTGCTGCTTACGGTACTGCTAAGTCTGGTGTTGGTATCTGTGCCACTTGTGTGTTGAGACCAGACCTATTATTCAAGAACATTGTTCCTGTTATTATGGCTGGTATCATTGCCATTTACGGTTTAGTTGTTTCCGTTTTGGTTTGTTATTCGTTGGGTCAAAAGCAAGCTCTGTACACCGGTTTCATCCAATTGGGTGCCGGTCTATCAGTCGGTTTGAGTGGTCTAGCTGCTGGTTTCGCTATTGGTATTGTCGGTGATGCAGGTGTTAGAGGTTCCTCTCAACAACCAAGATTATTCGTCGGTATGATTTTGATTTTGATTTTTGCTGAAGTTTTGGGTCTATACGGTTTGATTGTTGCTTTGTTGTTGAACTCCAGGGCTACTCAAGATGTTGTCTGTTAA |
| Rma1 | ATGGATGATATAAGCGGAAGGCAAACTTTACCTCGAATAAACCGTTTGTTGGAGCACGTGGGAAATCCCCAAGATAGTTTGTCAATCCTACATATAGCGGGAACAAATGGTAAGGAGACAGTATCTAAATTTTTGACATCCATATTGCAGCATCCGGGGCAACAGCGGCAAAGGGTCTTGATTGGTAGGTATACTACATCTTCCTTGCTAAACGCCAAAGAAGAGGATATTAGCATTAATAATGAAGCCATTTCCTTGATAGAGTATTCTAGGATCGAGAAGGAACTTATAGAAGCAGATAGTTCTTTGAAATTACAGTGCAACAACCTCGAACTGCTAACAAGCGTAGCTCTCGTATACTTCGCTAAGAAAAATTGCCAATGGTGCATAATAGAAACTGGTTTAGCTGGAAAACAGGACCCTGGAAGTATAATTGCTGGTCAAAGTAGAGTCTGTTGCGCCATTACTAACGTGGGCATTAGCGATGAAGCTTTTTTATGCAAGTTTTTGTCTCAAATTACTGAAAGTTCCACAAATAAAGCAATTTTTCTATTAGACGGTTCTAATGACGAATTTGTACGAAATACGATAACGAAACGGTGCCATGATGTTGGATGTCCATTAGAAATCACCGACCCTTCTCTTAGGGATTACAATGTACACACAGACACATGGGGCACTCTTGAAGTTCGCCTGCCATACAGTGAAGAAGAATATCAAATATTTAATTTGAGAGTTGCGATAGCGGTTTTAGACTTTTTGAGCAAGGAAAAAAAGGTTTGTATTTCAAAGGATCAACTATCCCAAGGTTTAATATCTGTGGATTGGCCAAGAAGTTTACATCGCTTGGATTACTGTTATGAATCTACTAGTGGAAAGAAAATCGCATTACTATTAGACAACGCAAATAATGCGAAGGCAGCTCGAAATTTAGCCTGCCATTTAAGGACCACGTACGGTGATACGCCATTAACATTTGTCATTGCTATAACAACTGGGAAAAAGGTGTCTCCCTTACTTGATCCGCTAATACGTCCACAAGATTATGTTATTGTGACTAGATTTGGGTCAGTGGTTGGAATGCCGTGGATCCAATCCCTAGAACCGGTGAATCTTCTCGCATTTATCAAAAACCGGTATACGAGAAATGTTAACATGCAGCCGGATCTTCAAAGTGTCTGGACCTTCCTTGAAACAAGTGGGTTAAAGACGATTGTTCCTGTTATCGTATGTGGATCACTGTATATCTGCAAAGAGCTATTGCGCTTACACAACTGTCACTTGCCAGTATAG |
| Hsp10 | ATGGCCGCCGAGGTGAAGACCGTGATCAAGCCCCTGGGCGACCGAGTGGTCGTGAAGCGAATCGAGGAAGAGCCCAAGACCAAGGGCGGCATCGTGCTGCCCGACACCGCCAAGGAGAAGCCTCAGAAGGGCAAGGTGATCGCCGTGGGCACCGGCCGAGTGCTGGAGAACGGACAGCGAGTGCCCCTGGAGGTGAAGGAGGGCGACATCGTGGTGTTCGCCAAGTACGGCGGCACCGAGATCGAGATCGACGGCGAGGAGTACGTGA TCCTGTCTGAGCGAGACCTGCTGGCCGTGCTGCAGTAA |
| Groes | ATGGAGGAGCTGGAGAAGGACAAGATCGAGCGAAACGAGGAGATGTCTGAAGAGGTGAAAGGCGAGGGCCCCCCCTCTGAGCTGGAGCAGTCTGAAGAGGTGGTGGAGGAGAAGATCGAGACCGAGGTGGAGCAGAAGAAGGAGCCCTCTCTGGAGGAGATCGTGGAGGAGCTGCGAAAGAAGCTGGAGGAGAAGGAGAAGGAGGCCAAGGAGTACCTGGACATCGCTCAGCGAATCAAGGCCGAGTTCGACAACTACCGAAAGCGAACCGAGAAGGAGAAGGCTGAGATGATCTCTTACGGCCAAGAGCAAGTGATCATCGAGCTGCTGCCCGTGATCGACAACTTCGAGCGAGCCCTGGCCAACGAGGGCGACTACAACTCTCTGCGAGAGGGCCTGGAGCTGATCTACCGACAGTTCAAGAAGGTGCTGGACAAGTTCGAGGTGCGAGAGATCGAGGCCGAGGGACAGATGTTCGACCCCTACAAGCACCACGCCCTGGCCCAAGAAGAGGTGGAGGGCAAGCAGCCCAACGAGATCATCGAGGTGTTTCAGAAGGGCTACTACCTGAAGGACAAGGTGATCCGACCCTCTCTGGTGAAGGTGGCCAAGTAA |
| Gros2 | ATGACCAAGATTCAGCCCGTGAACGGCCACGCCCTGATCAAGCTGGAGAAGGAGCCCGAGAAGAAGGTGGGCGGCGTGATCATCCCCAAGTCTGCCGAGGAGAAGCTGAATCAAGGCGTGATCGAGGCTATCGCCGCCGGCGCCACCGAGGAGCTGGCCGTGGGCGACCGAGTGATCTACAAGGAGTTCTCTGGCACCAAGATCAAGCACGAGGGCGAGGAGTACCTGATCATCCCCGTGGACGACATCCTGGCCAAGTTCGTGGAGGTGGACGAGATCTAA |
| Ibpa | ATGTCTCTGATGCGACGAGGCCGAGACTGGTGGGACTGGCCCTTCGACATCAACATCAGAAACCTGCCCTCTATCTTCGACATCAACTTCCCCTCTCTGTCTGGCCTGTTCTCTCGACCCCGAGTGGACATCGTGGAGTCTGAGACCGAGATCGTGGCCACCGCCGAGCTGCCCGGCGTGGACAAGAAGGACATCGAGATCAACGTGTACGACAACATCCTGGAGATCAAGGGACAGACCTCTGTGGACGAGGAGAAGGAGGACAAGAACTACTACATCCGAGAGCGATACTACGGCTCTTTCGCCCGACGAATCGAGCTGCCCGCCGAGGTGGACCCCGAGCGAACCACCGCCAAGTTCGAGAACGGCATCCTGAAGATCACCATGCCCAAGCTGCACCCCTCTAAGCCCAAGAAGCGACGAATCGACATCGAGTAA |
| Sso2427 | ATGCCCAAGCGAGAGGAGAAGGACATCTTCGACCTGATGGACGAGTGGATCCGAGAGATGGAGGAAGAGTTCGAGCGAATCGAGCGAGAGTTCATGCGAGGCTTCCGAGGCAAGGGCGAGGGCATCCGACAGTTCGGCCCCTACGTGTACGGCTTCCGAATCACCGTGGGCCCCGACGGCGTGCCCAAGATCGAGGAGTTCGGCAACGTGCGAAAGATCCGAGGCAAGCCCATGATCTCTGAGGAGCGAGAGCCCCTGGCCGACGTGATCGAGAAGGGCGACGAGATCAAGGTGGTGGCCGAGGTGCCCGGCGTGAACAAGGAGGACATCAAGGTGAAGGTGACCAACGGCGGCAAGAAGCTGGTGATCACCGCCAAGTCTGAGGACCGACAGTACTACAAGGAGATCGACCTGCCCGCCGAGGTGGACGAGAAGGCCGCCAAGGCCAACTTCAAGAACGGCGTGCTGGAGATCACCCTGAAGAAAAAGGCCTCTTCCTCTGACTCTGGCGTGGATATCAAGGTGGAGTAA |
| GCSGC | ATGACCCTGAATCAGCTGCTGCAGAAGCTGGAGGCCACCTCTCCCATCCTGCAAGCCAACTTCGGCATCGAGCGAGAGTCTCTGCGAGTGGACCGACAAGGACAGCTGGTCCATACCCCCCACCCCTCTTGTCTGGGCGCCCGATCTTTCCACCCCTACATTCAGACCGACTTCTGTGAGTTTCAGATGGAGCTGATCACCCCCGTGGCCAAGTCCACCACCGAGGCCCGACGATTCCTGGGCGCCATCACCGACGTGGCCGGCCGATCTATCGCCACCGACGAGGTGCTGTGGCCCCTGTCTATGCCCCCCCGACTGAAGGCCGAGGAGATTCAAGTGGCTCAGCTGGAGAACGACTTCGAGCGACACTACCGAAACTACCTGGCCGAGAAGTACGGCACCAAGCTGCAAGCCATCTCTGGCATCCACTACAACATGGAGCTGGGCAAGGACCTGGTGGAGGCCCTGTTCCAAGAGTCTGATCAGACCGACATGATCGCCTTCAAGAACGCCCTGTACCTGAAGCTGGCTCAGAACTACCTGCGATACCGATGGGTGATCACTTACCTGTTCGGCGCCTCTCCCATCGCCGAGCAAGGCTTCTTCGACCAAGAGGTGCCCGAGCCCGTGCGATCTTTCCGAAACTCTGACCACGGCTACGTGAACAAGGAGGAGATCCAAGTGTCTTTCGTGTCTCTGGAGGACTACGTGTCTGCCATCGAGACCTACATCGAGCAAGGCGACCTGAACGCCGAGAAGGAGTTCTACTCTGCCGTGCGATTCCGAGGCCAAAAAGTGAACCGATCTTTCCTGGACAAGGGCATCACCTACCTGGAGTTCCGAAACTTCGACCTGAACCCCTTCGAGCGAATCGGCATCTCTCAGACCACCATGGACACCGTGCACCTGCTGATCCTGGCCTTCCTGTGGCTGGACTCTCCCGAGAACGTGGACCAAGCCCTGGCCCAAGGCCACGCCCTGAACGAGAAGATCGCCCTGTCTCACCCCCTGGAGCCCCTGCCCTCTGAGGCCAAGACCCAAGACATCGTGACCGCCCTGGATCAGCTCGTGCAGCATTTCGGCCTGGGCGATTACCACCAAGACCTGGTGAAGCAAGTGAAGGCCGCCTTTGCCGACCCCAATCAGACCCTGTCTGCTCAGCTGCTGCCCTACATCAAGGACAAATCTCTGGCCGAGTTCGCCCTGAACAAGGCCCTGGCCTACCACGACTACGACTGGACCGCCCACTACGCCCTGAAGGGCTACGAGGAGATGGAGCTGTCTACTCAGATGCTGCTGTTCGACGCCATTCAGAAGGGCATCCACTTCGAGATCCTGGACGAGCAAGATCAGTTCCTGAAGCTGTGGCACCAAGACCACGTGGAGTACGTGAAGAACGGCAACATGACCTCTAAGGACAACTACGTGGTGCCCCTGGCCATGGCCAACAAGACCGTGACCAAGAAGATCCTGGCCGACGCCGGCTTCCCCGTGCCCTCTGGCGACGAGTTCACCTCTCTGGAGGAGGGCCTGGCCTACTACCCCCTGATCAAGGACAAGCAAATCGTGGTGAAGCCCAAGTCTACCAACTTTGGCCTGGGCATCTCTATCTTCCAAGAGCCCGCCTCTCTGGACAACTATCAGAAGGCCCTGGAGATCGCTTTCGCCGAGGATACCTCTGTGCTGGTGGAGGAGTTCATCCCCGGCACCGAGTACCGATTCTTCATCCTGGACGGCCGATGTGAGGCCGTGCTGCTGCGAGTGGCCGCCAACGTGATCGGCGACGGCAAGCACACCATCCGAGAGCTGGTGGCTCAGAAGAACGCCAACCCCCTGCGAGGCCGAGACCACCGATCTCCCCTGGAGATCATCGAGCTGGGCGACATCGAGCAGCTGATGCTGGCTCAGCAAGGCTACACCCCCGACGACATCCTGCCCGAGGGCAAGAAGGTGAACCTGCGACGAAACTCTAACATCTCTACCGGCGGCGACTCTATCGACATCACCGAGACCATGGACTCTTCTTACCAAGAGCTGGCCGCTGCCATGGCCACCTCTATGGGCGCCTGGGCCTGTGGCGTGGACCTGATCATCCCCGACGAGACTCAGATCGCCACCAAGGAGAACCCCCACTGTACCTGTATCGAGCTGAACTTCAACCCCTCTATGTACATGCACACCTACTGTGCCGAGGGCCCCGGCCAAGCCATCACCACCAAGATCCTGGACAAGCTGTTCCCCGAGATCGTGGCCGGACAGACCTAA |
| Cspl | ATGGAGCACGGCACCGTGAAGTGGTTCAACTCTGAGAAGGGCTACGGCTTCATCGAGCGAGAGGGCGGCGACGACGTGTTCGTGCACTTCTCTGCCATCCAAGGCGAGGGCTACAAGACCCTGGAGGAGGGACAGAAGGTGTCTTTCGACGTGGAGGAGGGCTCTCGAGGCCCCCAAGCCGCCAACGTGCAGAAGGAGGACTAA |
| Hsf1 (KM) | ATGGACGAGATCACCAATCCTAACGACATCCTGCTGCCCCACGAGGGCACCGGCCTGACCCCCGTGGGCATCTCTGTGGGCCCCGAGGCCTACCCCATCAACGACGTGCTGCACACCTCTCACCTGAAGGGCGCCGCCGACGTGGAGGACCCCCTGATCGAGGACATCGTGAACCCCTCTCTGGACCCCGAGGGCTCTTCCTCTGAGCCCTCTAACGAGGTGGTGACCCCCCTGATGCACCCCTCTTTCAACATCGACCACAACATGCACCGACCCTCTCCCGTGACCGGCGTGTACGGCCTGGCCAACACCCCCGGCAACCAAGACTACAAGTCTACCACTACCGAGTCTTCTCGACGAGAGTCTTTCCTGAACGACGCCGACAATCAGCAGAATCAGCACACTCAGCCCGGCAACCGAGGACAGAAGTACTACAACGGCTACTCTCAGACCCACCATCAGCCCGTGGTGTCTCTGAACAAGCGAAAGCTGCTGGCCAAGCCCCACGGCGTGGACAAGCCCCACTCTAAGAAAAAGATGTCTACCACCCGAGCCCGACCCGCCTTCGTGAACAAGCTGTGGTCTATGGTGAACGACTCTACCAACGAGAAGTTCATCCACTGGTCTGACTCTGGCGAGTCTATCGTGGTGCCCAACCGAGAGCGATTCGTGCAAGAGGTGCTGCCCAAGTACTTCAAGCACTCTAACTTCGCCTCTTTCGTGCGACAGCTGAACATGTACGGCTGGCACAAGGTGCAAGACGTGAAGTCTGGCTCTATGCTGTCTAACAACGACTCTCGATGGGAGTTCGAGAACGAGAACTTCAAGAAGGGCAAGGAGAACCTGCTGGAGAACATCGTGCGACAGAAGCCCAACTCTAACATGGTGGGCGGAGGCAATGACGAGGTGGACATCAACATCCTGATGAACGAGCTGGAGACCGTGAAGTACAATCAGCTGGCCATCGCCGAGGACCTGCAGCGAATCACCAAGGACAACGAGATGCTGTGGAAGGAGAACATGATGGCCCGAGAGCGACATCAGTCTCAGCAACAAGTGCTGGAGAAGATCCTGCGATTCCTGTCTTCTGTGTTCGGCCCCAACTCTGCCAAGACCATCGGCAACGGCTTTCAGCCCGACCTGATCCACGAGCTGGGCGACATGCAAGTGCAGTCTCCCAACAATAACACCGCCAACATGAACCCCGGCTCTTACCACAACGAGGAGGACCCCATGGCCAACGTGTTCGGCCCCCTCACCCCCTCTGATCAGATGAAGATCCCTCAGAACCAAGACCACAAGGTGCGACCCCGACTGCTGATCAAGAACCGATCTGGCTCTTCTTCCTCTTCCTCTATTCCCTCTGAGCGAGCCTCTTCTTACCACCGAATGGTGTCTACCCCCGTGGCCGTGGATCAGCTGCATCAGCAGCACACTCAGCAGCAACAGCAACAGCAACAGCCCCACATTCAGCAGCGAAAACAAAGACAAGCTCAGCCTCAGTTCCCCTACGTGATTCAGAACTCCAACGGCAATTCTAACGGCAACGGCAACGGCTCTGCCAACCCCGCCATCAACAACATCGGCGGAGGCAACGGCACCCAAGTGGGCGTGCAGACCTTTCAGCCTCAGCACTCTATCCACTCTTCTAACGGCGACATCCAAGAGCTGACTCCCTCTATTATCTCTTCTGACTCTCCCGACCCCTCTTTCTTCCAAGACCTGCAGAACAACATCGACCGACAAGAGGAGTCTATCCAAGAGATCCAAGACTGGATCACCAAGCTGTCTCCCGGCGAGGACGGCAACACCCCCATCTTCCCCGACCTGAACATGGCCTCTCAGTTCACCACCTCTCACGGCAACCAAGGACAGATCCCCTCTCAGACCCCCGACTTCAACAACTCTCACATCGAGGAGCTGCAGCAGCCCCGACTGAACGAGCCCGACATCCCTTCTGAGGAGTTCAACCCTCAGCGAAAGCGACGAAAAGCCACCTAA |
| Msn2 (KM) | ATGACCCTGGGCGGCTTCACCTCTGAGAACTCTCTCGACATCCGATCTGACTCTATGTCTGGCATCTACGGCGAGCGATCCCAAGTGGAAGCCGGCGGAGGCGACATCGGCGAGGGCGGCACCCCCGCCATCAAGAACGTGAAGCTGGAGACCCTGCAGCACAACATGGGCTCTACCCCCACCGAGATGAACGACTTCCTGGCCATGCTGGACGACAAGACCACCTACTCTGAGGTGATTCAGCTGACCGAGCCCAACATGTCTTCTGAGCGACCTCAGTCTATGGACTACAACACTGGCTCTGAACTGTCTTTCAACGGCCGAGACATGATCGGCGCCGGCTCTCCCATGGGCTCTTACCCCAACTCTCAGCGAATCTCTAACCGATCTTCTCGAGACCACACCCTGAACTACTCCGAGCCCGAACCCGGCGACTCCGGCGAGCTCGGCGAGACCATGGACCCCCTGACCGCCATCGCCTCTGCCTCTATCTCTTCTGTGCCTGGCTCTGAGCAGCACTCTCGAGTGCCCCAAGTGCAGCGAATGACTCAGCAGCCTCAGACCACCGAGAACTCTATGCAGAAGACCACCCTGAACTCTACCTCTATCCAAGACTTCCTGGACAACATCGACAACTCTCAGTCTGAGGAGCAGTACATCAACCCCTACCTGCTGAACAAGGAGATGAACGGCGCCGGCGTGGGCCCCTCTCTGTTCATGGACTCTGGAACCGACATCGACAACAACAAGACCAACGACGACACCAACCCCATCTCTGAGAACCCCATGATGCTGGAGGACCTGACCGTGTCTCCTCAGCCCGTGTTCTCTGACCGAAGACGAATGTCTGAGGTGGCCTCTGAGAAGGTGGGCTACCCCTCTGGCCCCCGAGGCTCTATCTCTCACCAAGTGGACTTCTGGAACCTGTCTTCTGCCGGCTCTTCTAACCCCCCCCTGCTGAACATGTCTCCCAACAAGTCTGCTCAGCTGCAGCAAGAGAACAACTCTGAGCTGTTCGACCTGATGTCTTTCAAGAAAAAGGGCCGACAGCAGTCTCTGCAGCAGCAAAAGATGCATAACCCCACCACTCAGCAACAGAAGGACCTGCAGCACGGCGACCTGCAGCAGACTCAGCAACAGCAACAGCAACAACAACAGCAACAGCAGCGACAGTCTGCCTTCAAGATCGACAACGAGCTGACTCAGCTGCTGAACGCCTACAATCTCTCTCAACCCAACGTGACCGCCCGAAACGCCAACAACATCAACAATAACTCTGGCACCGCCACCAAGATGCGAACCGGCTCTTTCAACCAAGCCAACATCAAGCGATCTAACTCTTCTACCCAAGAGGCCCACAACCGAGTGGGCAAGCAGCGATACTCTATGTCTCTGCTGGACGGCAACCAAGACGCCATCTCTAAGCTGTACGGCGACATGGCCCGAAACGGCATGTCTTGGGAGAACGCCATCATCTCTGACGACGAGGAAGAGCGAGACCGACAAGACGACACTCAGAAGGTGCGACGAAAGTCTTCTCTGAACCGAAAGAACCAAGAGGTGTCTCAGTCTTCTGTGGACGCCACCGGCGGCCGATTCATCTCTCCTCAGCTGCTGAATAACGACCCCCTGCTGGAGAATCAGCTGAACGCCGGACAGAACTCTCTGGGAGTGGACCGATCTAACCTGAACTTCGAGCTGAACCTGCCCATCACCAACCCCGAGGCCCTGATCGGCGCCACCTCTCCCAATTCTATGACCACCTCTTCTAACGTGTCTTCTACCGGCTACAACTCTGCCGGCGCCGTGGCCGGCCCCGCCTCTGGACAGCGACGAAAGCGACTGTCTATGTCTAAGCCCAAGGTGATGACCAAGGCCACCTCTCCCCTGGACGGCGAGGAGAAGCCCTTCAAGTGTGATCAGTGTTCTAAGACCTTCCGACGATCTGAGCACCTGAAGCGACACGTGCGATCTGTGCACTCTACCGAGCGACCCTTCCACTGTCAGTTCTGTGACAAGAAGTTCTCTCGATCTGACAACCTGTCTCAGCACCTGAAGACCCACAAGAAGCACGGCGACATCACCGAGCTGCCCCCTCCCCGACGAGCCACCAACTCTTCCTCTAAGTAA |
| SHsp | ATGGTGCGACGAATCCGACGATGGGACATCTGGGACCCCTTCGACCTGATCCGAGAGATCCAAGAGGAGATCGACGCCATGTTCGACGAGTTCTTCTCTCGACCCCGACTGTGGACCTACCGACGATGGTCTGAGCCCGCCATGTACGAGGAGCGAGTGGGCGAGGTGTGGCGAGAGCCCTTCGTGGACATCTTCGACAACGGCGACGAGTTCGTGATCACCGCCGAGCTGCCCGGCGTGCGAAAGGAGGACATCAAGGTGCGAGTGACCGAGGACACCGTGTACATCGAGGCCACCGTGAAGCGAGAGAAGGAGCTGGAGCGAGAGGGCGCCGTGCGAATCGAGCGATACTTCACCGGCTACCGACGAGCCATCCGACTGCCCGAAGAGGTGATCCCCGAGAAGGCCAAGGCCAAGTACAACAACCAAGTGCTGGAGATCCGAGTGCCCAAGAAGCACCCCACCAAGAAGGAGTCTGAGGGCTTCGAGGTGAAGGTGGAGTAA |
| Tte2469 | ATGACCGTGATCTTCGTGGGCAAGGACAATCAGATCACCATCAAGGCCCCCAAGAACGTGGAGAAGCTGGCCAAGGAGCTGGACATCAACCTGGAGTCTCACGTGTTCATCAAGAACGGCGAGATCGTGACCCCCGACGAGATCCTGCAAGACGATGACGTGGTGGAGATCATCTC TGTGGTGTCTGGCGGCTAA |

**
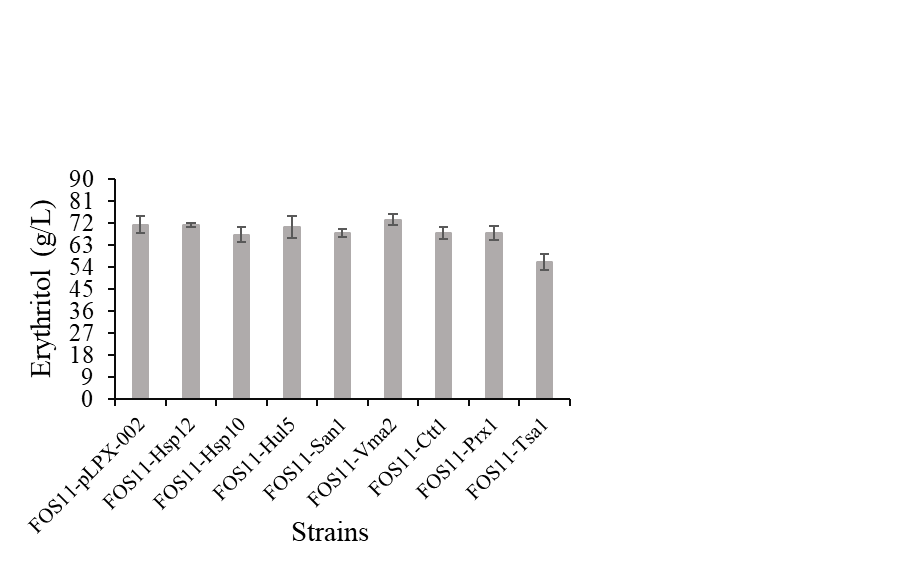
**

**Figure S1**. Erythritol production by engineered strains at 30 ^o^C.


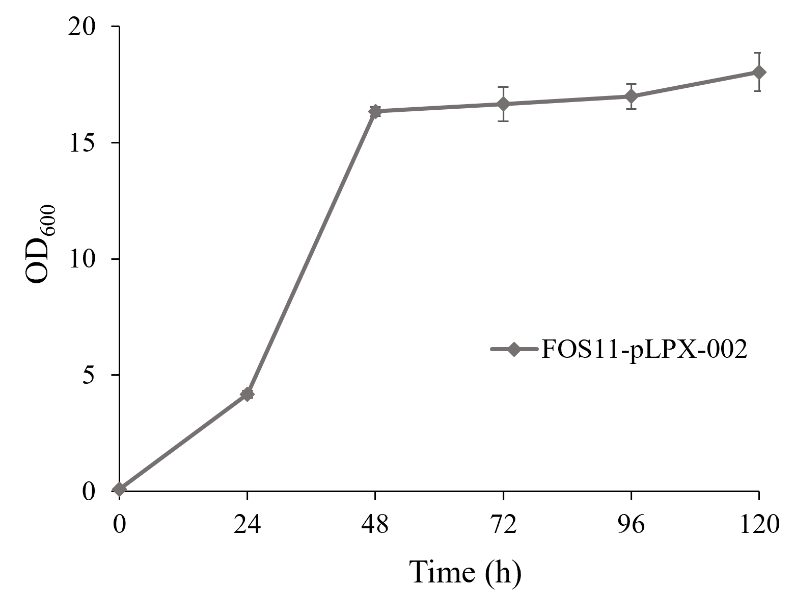


**Figure S2**. The growth cycle of strain FOS11-pLPX-002 at 35 ^o^C.

**
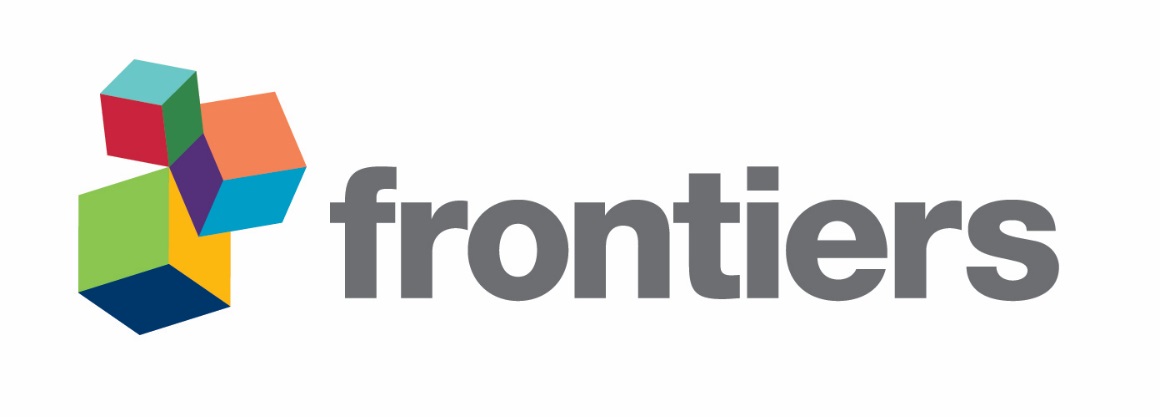
**
